# The crystal structures determination and Hirshfeld surface analysis of *N*-(4-bromo-3-meth­oxy­phen­yl)- and *N*-{[3-bromo-1-(phenyl­sulfon­yl)-1*H*-indol-2-yl]meth­yl}- derivatives of *N*-{[3-bromo-1-(phenylsulfon­yl)-1*H*-indol-2-yl]meth­yl}benzene­sulfonamide

**DOI:** 10.1107/S2056989024009587

**Published:** 2024-10-04

**Authors:** S. Madhan, M. NizamMohideen, Vinayagam Pavunkumar, Arasambattu K. MohanaKrishnan

**Affiliations:** a Department of Physics The New College Chennai 600 014 University of Madras,Tamil Nadu India; b Department of Organic Chemistry University of Madras, Guindy Campus Chennai-600 025 Tamilnadu India; National Taras Shevchenko University of Kyiv, Ukraine

**Keywords:** crystal structure, 1*H*-indole, benzene­sulfonamide, π–π inter­actions, hydrogen bonding, Hirshfeld surface analysis

## Abstract

Two new phenyl­sulfonyl­indole derivatives, namely, *N*-{[3-bromo-1-(phenyl­sulfon­yl)-1*H*-indol-2-yl]meth­yl}-*N*-(4-bromo-3-meth­oxy­phen­yl)benzene­sulfonamide, C_28_H_22_Br_2_N_2_O_5_S_2_, (**I**), and *N*,*N*-bis­{[3-bromo-1-(phenyl­sulfon­yl)-1*H*-indol-2-yl]meth­yl}benzene­sulfonamide, C_36_H_27_Br_2_N_3_O_6_S_3_, (**II**), reveal the impact of intra­molecular π–π inter­actions of the indole moieties as a factor not only governing the conformation of *N*,*N*-bis­(1*H*-indol-2-yl)meth­yl)amines, but also significantly influencing the crystal patterns. For **I**, the crystal packing is dominated by C—H⋯π and π–π bonding, with a particular significance of mutual indole–indole inter­actions. In the case of **II**, the mol­ecules adopt short intra­molecular π–π inter­actions between two nearly parallel indole ring systems [with the centroids of their pyrrole rings separated by 3.267 (2) Å] accompanied by a set of forced Br⋯O contacts. This provides suppression of similar inter­actions between the mol­ecules, while the importance of weak C—H⋯O hydrogen bonding to the packing naturally increases. Short contacts of the latter type [C⋯O = 3.389 (6) Å] assemble pairs of mol­ecules into centrosymmetric dimers with a cyclic *R*_2_^2^(13) ring motif. These findings are consistent with the results of a Hirshfeld surface analysis and together they suggest a tool for modulating the supra­molecular behavior of phenyl­sulfonyl­ated indoles.

## Abstract 2

The crystal structures of two 1*H*-indole derivatives are described and the analysis of the inter­molecular contacts in the crystals using Hirshfeld surface analysis and two-dimensional fingerprint plots is reported.

## 1. Chemical context

Sulfonamide derivatives found applications in modern medicine to control diseases caused by bacterial infections (Brown, 1971 ▸; Zhao *et al.*, 2016 ▸). These species have been famous as sulfa drugs for over 70 years since the discovery of their activity. They are still used as anti­biotics (Gulcin & Taslimi, 2018 ▸), in spite of the later introduction of penicillin. In particular, numerous formulations based on sulfonamides have repeatedly been used as chemotherapeutics for their anti­bacterial (Ovung & Bhattacharyya, 2021 ▸; Badr, 2008 ▸), anti­fungal (Hanafy *et al.*, 2007 ▸) and hypoglycemic properties (Chohan *et al.*, 2010 ▸; El-Sayed *et al.*, 2011 ▸). Among drugs of other types, sulfonamides also display appreciable anti­tumor, anti­cancer, and anti­thyroid activities (Scozzafava *et al.*, 2003 ▸). Some sulfonamide products also possess carbonic anhydrases (CA) inhibition properties (Suparan *et al.*, 2001 ▸). The production of new compounds with noteworthy biological activity, which are suited as anti­viral and anti­microbial agents, drives inter­est in synthetic approaches for sulfonamide-functionalized heterocyclic ring systems (Azzam *et al.*, 2020 ▸). Identifying the significance of such compounds for biochemical uses and drug discovery, and our continuing study of the development of indole products have prompted us to examine a series of corresponding *N*-sulfonyl- and bromo-substituted species. The need for deeper functionalization of the systems by introducing bromine substitutes is motivated by the fact that the presence of halogen atoms in mol­ecules commonly enhances various biological activities and thus halogenation may be recognized as an essential tool for drug optimization (Murphy *et al.*, 2003 ▸). For example, the occurrence of bromine atoms on a phenol ring is important for improved anti­microbial activity (Bouthenet *et al.*, 2011 ▸). Structural trends in such compounds, including subtle features of their inter­molecular inter­actions, could be applicable to the specific targeting of the substrates in biomedical systems and therefore they may provide new insights into the action of sulfonamide derivatives. In particular, Adsmond & Grant (2001 ▸) categorized the hydrogen-bonding preferences of sulfonamides. The availability of multiple aromatic groups in *N*-sulfonyl­ated indoles imposes also possibility for versatile stacking patterns, which may be competitive to conventional hydrogen bonding. We report herein the crystal-structure determination and Hirshfeld surface analysis of two new indoles: namely, *N*-{[3-bromo-1-(phenyl­sulfon­yl)-1*H*-indol-2-yl]meth­yl}-*N*-(4-bromo-3-meth­oxy­phen­yl)benzene­sulfonamide, C_28_H_22_Br_2_N_2_O_5_S_2_, (**I**), and *N*,*N*-bis­{[3-bromo-1-(phenyl­sulfon­yl)-1*H*-indol-2-yl]meth­yl}benzene­sulfonamide, C_36_H_27_Br_2_N_3_O_6_S_3_, (**II**), which feature a complex inter­play of weak hydrogen-bonding and π–π inter­actions.
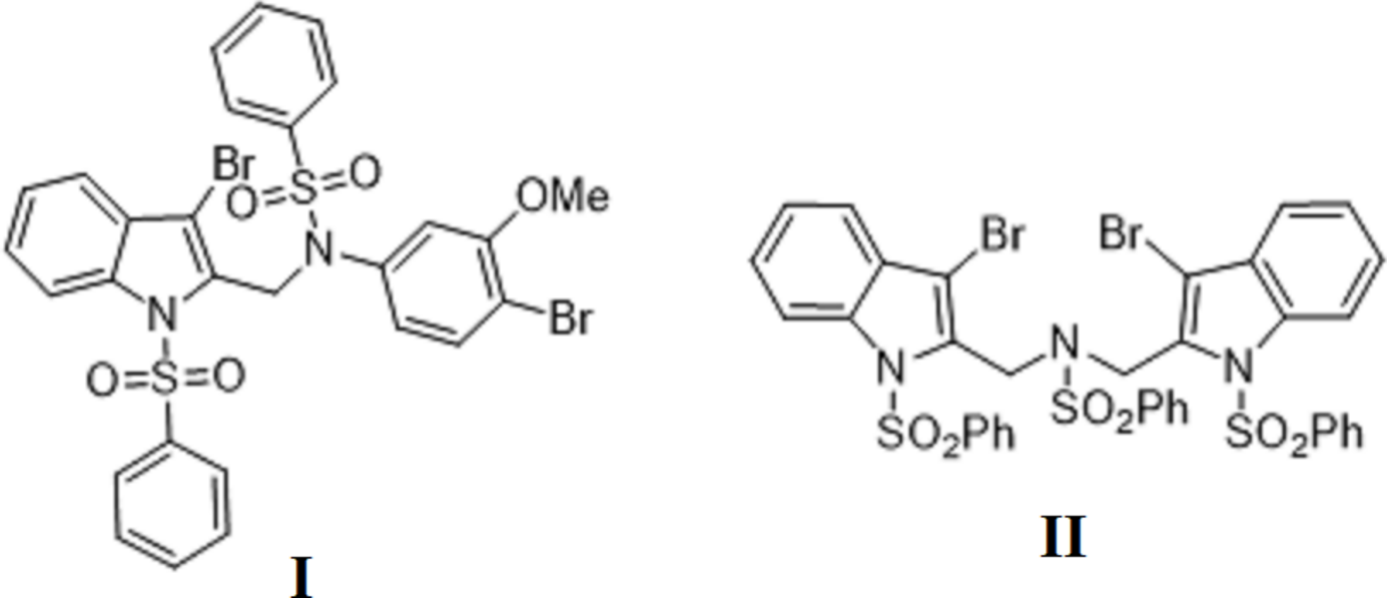


## 2. Structural commentary

**Figure 1:**
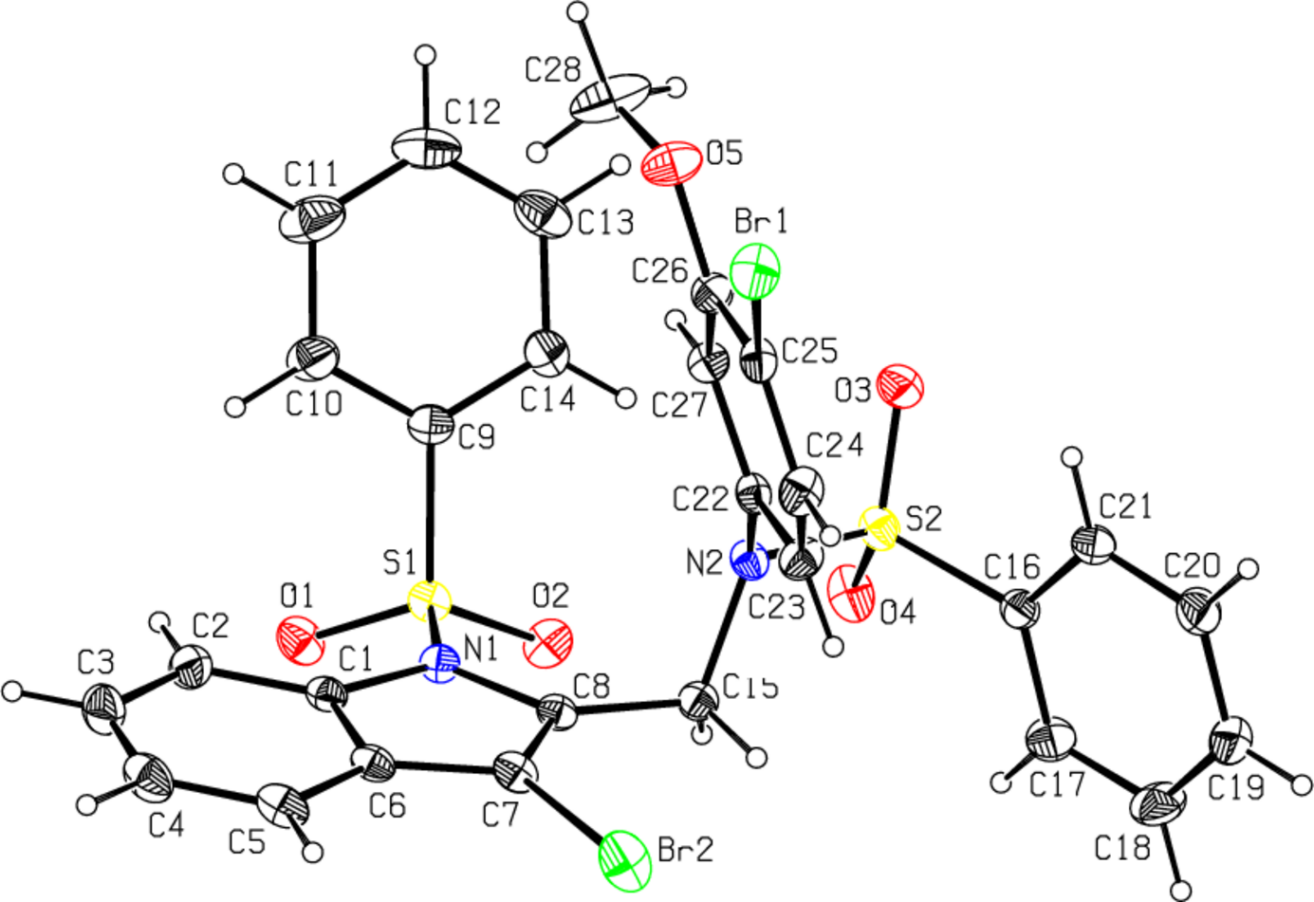
The mol­ecular structure of compound **I**, with atom labeling and displace­ment ellipsoids drawn at the 20% probability level.

The mol­ecular structures of the title compounds, which differ in substituents at the phenyl­sulfonyl­ated exocyclic N2 atoms (*N*-(4-bromo-3-meth­oxy­phen­yl) for **I** and *N*-{[3-bromo-1-(phenyl­sulfon­yl)-1*H*-indol-2-yl]meth­yl} for **II**), are illustrated in Figs. 1 ▸ and 2 ▸, respectively. In both the cases, the indole ring systems (N1/C1–C8 and N3/C23–C30) are essentially planar, with a maximum deviation from the corresponding mean planes of 0.039 (4) Å observed for C8 atom in **II**. The torsion angles involving the sulfonamide fragments, O2—S1—N1—C1 [−152.6 (3) for **I** and −175.2 (3)° for **II**], O3—S2—C16—C17 [−153.9 (3) for **I** and −146.4 (3)° for **II**] and O5—S3—N3—C23 [−178.7 (3)° for **II**] indicate an anti-periplanar conformation of the sulfonyl moiety. The dihedral angle between sulfonyl-bound phenyl rings (C9–C14) and the carrier indole ring systems (N1/C1–C8) are 62.0 (2)° for **I** and 70.9 (2)° for **II**, unlike the orthogonal orientation of these groups in previously reported *N*-phenyl­sulfonyl indoles (Madhan *et al.*, 2022 ▸, 2023*a* ▸,*b* ▸, 2024*a* ▸,*b* ▸). In **I**, the dihedral angle between two sulfonyl-bound phenyl rings (C9–C14 and C16–C21) is 59.0 (2)°, while in **II** they are nearly orthogonal [86.5 (2)°]. The meth­oxy-bound phenyl ring (C22–C27) in **I** is also inclined to the indole framework, subtending a dihedral angle of 73.23 (1)°.

The geometric parameters of **I** and **II** agree well with those reported for related structures (Madhan *et al.*, 2022 ▸, 2023*a* ▸,*b* ▸, 2024*a* ▸,*b* ▸). The sulfonamide S atoms exhibit a distorted tetra­hedral geometry with the O—S—O angles lying in the range of 119.9 (2)–120.3 (2)°. The increase in these angles, accompanied by a simultaneous decrease in the N—S—C angles [which are 104.3 (2)–106.1 (2)°], from the ideal tetra­hedral values are attributed to the Thorpe–Ingold effect (Bassindale, 1984 ▸). The widening of the angles may be due to the repulsive inter­action between the two short S=O bonds. In both compounds, the expansion of the *ipso* angles at atoms C1, C3, C4 (and C25, C27, C28 in **II**), together with the contraction of the apical angles at atoms C2, C5, C6 (and C26, C29, C30 in **II**) are caused by fusion of the smaller pyrrole ring with the six-membered benzene ring and the strain is taken up by the angular distortion rather than by bond-length distortion (Allen, 1981 ▸).

The mol­ecular conformation of compound **I** is stabilized by the weak intra­molecular hydrogen bond C2—H2⋯O1 [C2⋯O1 = 2.908 (5) Å] formed by the sulfone O atoms, which generates an *S*(6) ring motif. The similar inter­action in compound **II** [C2⋯O1 = 2.993 (7) Å] is accompanied by three additional intra­molecular hydrogen bonds involving methyl­ene donors and sulfone acceptors [C15⋯O2 = 2.850 (5) Å, C22⋯O6 = 2.950 (5) Å and C29⋯O5= 2.907 (5) Å], which generate *S*(6) ring motifs.

**Figure 2:**
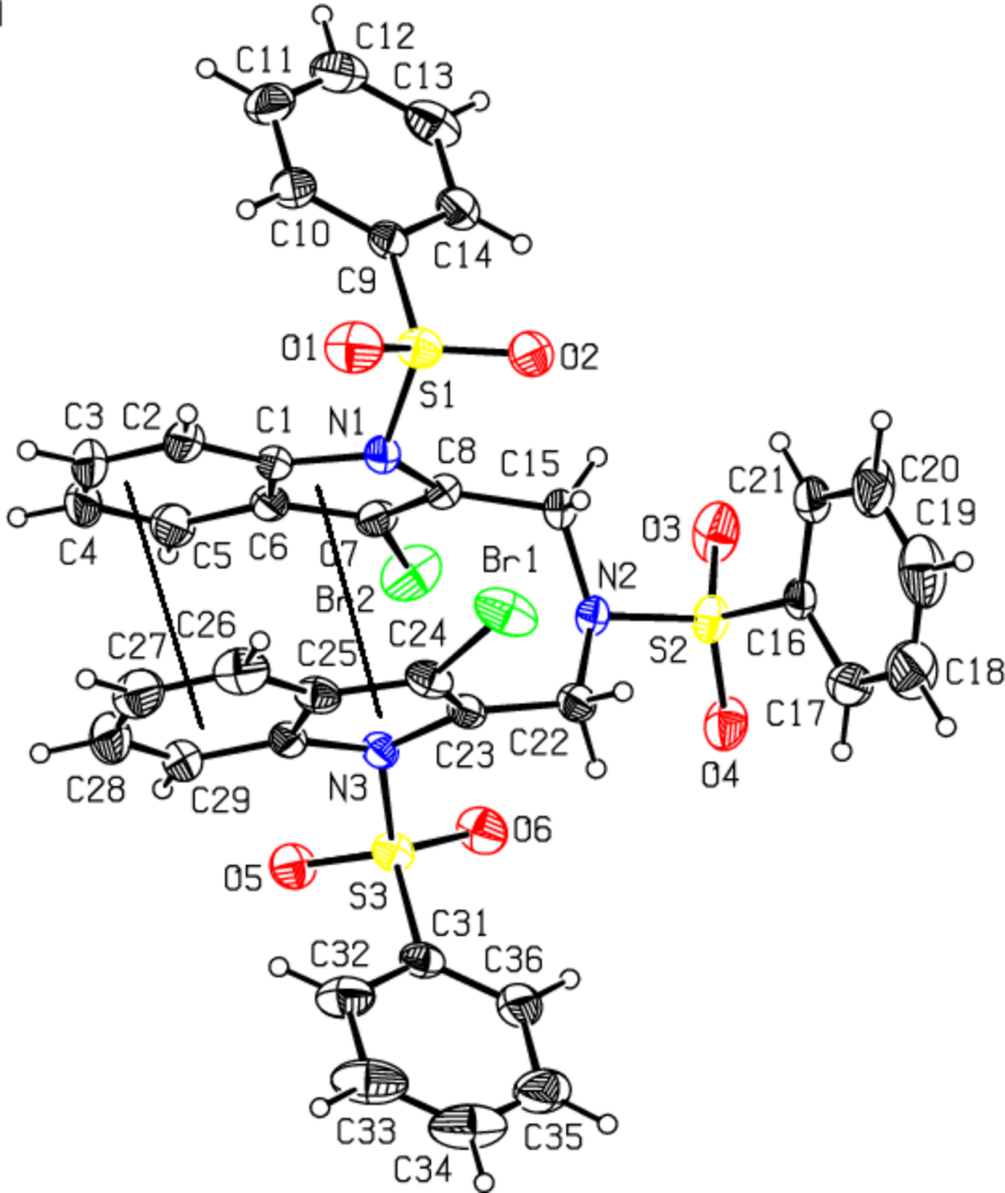
The mol­ecular structure of compound **II**, with atom labeling and displacement ellipsoids drawn at the 30% probability level. The dashed lines indicate the intra­molecular π–π inter­actions of the indole ring systems.

The most striking feature of the mol­ecular structures is the specific conformation of **II**, which is controlled by an intra­molecular π–π inter­action between the two indole ring systems (Fig. 2 ▸). Their planes are almost parallel, while adopting a small angle of 7.2 (2)°. Two pyrrole and two benzene rings are situated one on the top of another, with the corresponding inter­centroid distances being 3.267 (5) and 3.593 (5) Å, respectively, and with the shortest contact of 3.035 (5) Å observed between atoms C8 and C23. A similar intra­molecular pairing of aromatic rings separated by flexible triatomic spacers is relevant for the appropriate model of di­benzyl­ketone (Lima *et al.*, 2010 ▸). For the latter, the stacked conformation was associated with a relatively small stabilizing enthalpic effect of about 12.9 kJ mol^−1^ and therefore the crystal structure did not inherit the intra­molecular stacking observed for the gas phase and solution structures. In contrary, the energetics of the intra­molecular indole–indole inter­action could be estimated to be far superior (up to 50–60 kJ mol^−1^; Madhan *et al.*, 2024*a* ▸) due to the significantly larger inter­action areas and higher contribution of London dispersion forces. The impact of the resulting intra­molecular stacking on the sulfonyl­indole geometry is visible from the inspection of configuration around sulfonyl N atoms. The sum of the bond angles around N1 in **I** [359.5 (2)°] indicates *sp*^2^ hybridization (Beddoes *et al.*, 1986 ▸). However, in the case of **II**, two such parameters are essentially smaller [346.6 (2)° and 349.9 (2)°, for N1 and N3, respectively] and therefore these indole N atoms are pyramidalized to almost the same extent as the exocyclic sulfonamide N2 atoms [347.8 (2)° in **I** and 342.2 (2)° in **II**]. The configuration for the latter is typical for sulfonamides, which lack π-bonding between the N and S atoms (Blahun *et al.*, 2020 ▸). The perceptible pyramidalization of the indole N atoms may be viewed as a consequence of the steric strain imposed by close contacts between the Br and O atoms of two stacked indole systems [the shortest contact is Br2⋯O6 = 3.592 (4) Å]. At the same time, as a result of the electron-withdrawing character of the phenyl­sulfonyl group, the indole N—C*sp*^2^ bond lengths [N1—C1 = 1.415 (4) and N1—C8 = 1.427 (4) Å in **I**; 1.427 (5)–1.430 (4)Å in **II**] are longer than the mean value of 1.355 (14) A° for this bond (Allen *et al.*, 1987 ▸; Cambridge Structural Database (CSD), Version 5.37; Groom *et al.*, 2016 ▸).

## 3. Supra­molecular features

**Figure 3:**
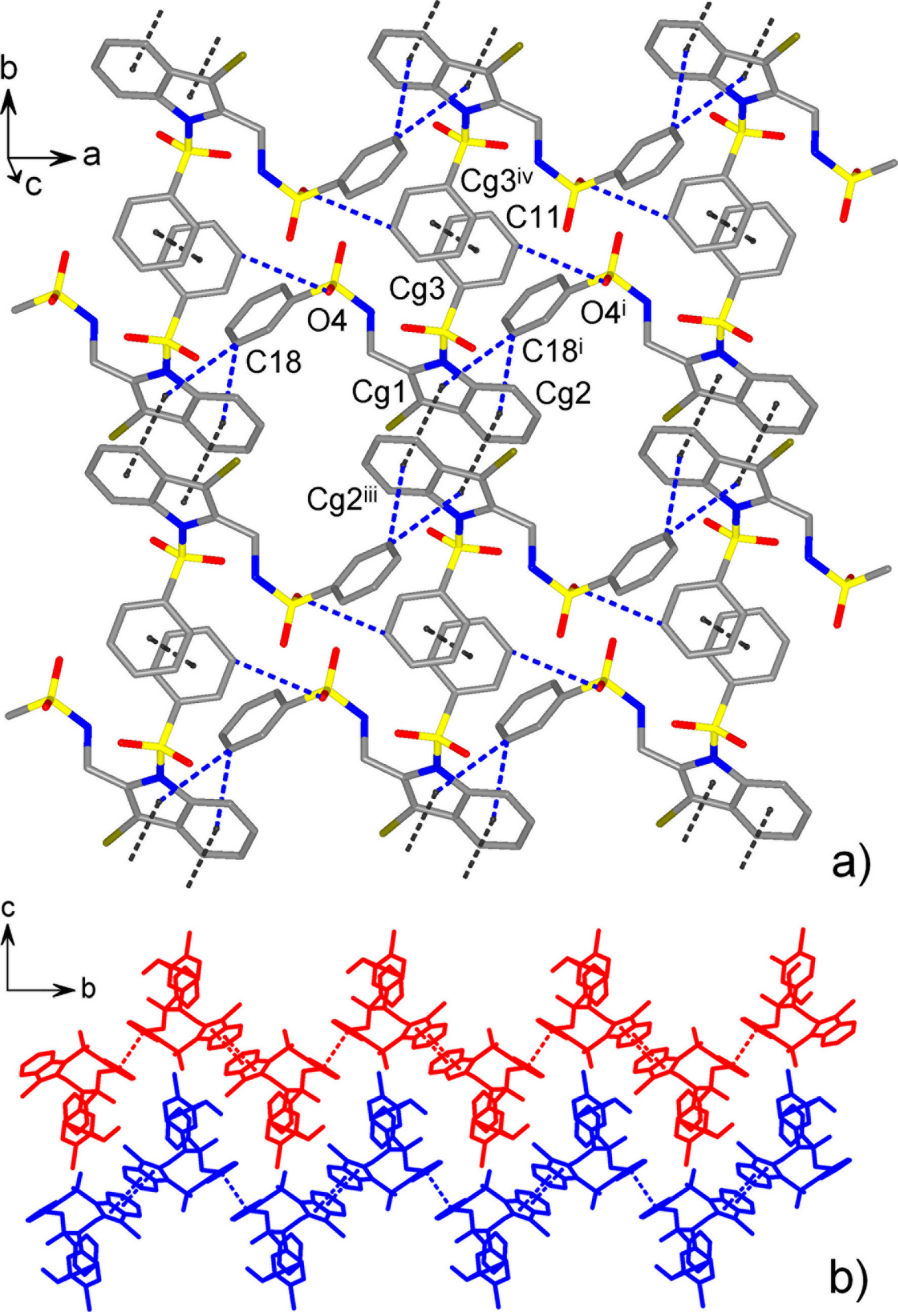
(*a*) Crystal packing of compound **I**, viewed in a projection nearly on the *ab* plane, showing a non-covalent layer assembled by π–π and C—H⋯O inter­actions (identified by dotted lines). (*b*) Packing of two successive corrugated layers. [Symmetry codes: (i) *x* + 1, *y*, *z*; (iii) −*x* + 1, −*y* + 1, −*z* + 1; (iv) −*x* + 1, −*y* + 2, −*z* + 1.]

With the absence of conventional hydrogen-bond donor functionality, the supra­molecular patterns of both compounds are controlled by weaker inter­actions, namely by weak C—H⋯O, C—H⋯Br and C—H⋯π hydrogen bonds (Tables 1 ▸ and 2 ▸) and slipped π–π stacking inter­actions (Table 3 ▸). In the case of **I**, the latter is prevalent. Anti­parallel stacking of two inversion-related indole ring systems [symmetry code: (iii) −*x* + 1, −*y* + 1, −*z* + 1) assemble the mol­ecules into dimers, which are connected into chains along the *b*-axis direction by means of π–π inter­actions between inversion-related phenyl rings [symmetry code: (iv) −*x* + 1, −*y* + 2, −*z* + 1] (Fig. 3 ▸). For the indole–indole inter­action, the corresponding inter­centroid distances of 3.532 (2) Å and shortest contacts, down to 3.456 (2) Å (Table 3 ▸), are consistent well with those for π–π inter­actions seen in the crystal structures of similar 1-(phenyl­sulfon­yl)-1*H*-indole derivatives (Madhan *et al.*, 2024*a* ▸). Further connection of the chains by C—H⋯π hydrogen bonds yields corrugated layers parallel to the *ab* plane. Two such C—H⋯π inter­actions actualize above and below the indole-indole stacks and they are bifurcated, involving both benzo- and pyrrole rings as the acceptors. The corresponding separations are C18⋯*Cg*(N1/C1/C6–C8)^ii^ = 3.861 (8) Å and C18⋯*Cg*(C1–C6)^ii^ = 3.579 (1) Å [symmetry code: (ii) *x* − 1, *y*, *z*). The only C—H⋯O bond in the structure [C11⋯O4^i^ = 3.503 (6)Å; symmetry code: (i) *x* + 1, *y*, *z*] is also identified withing this layer.

**Figure 4:**
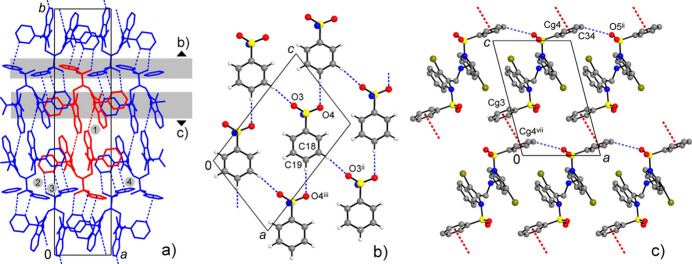
(*a*) Projection of the structure of **II** on the *ab* plane, showing the assembly of centrosymmetric C—H⋯O-bonded dimers and their integration into the three-dimensional framework. An individual dimer is colored red and its principal inter­actions C27—H⋯O1^iv^, C13—H⋯O3^i^, C14—H⋯O4^i^ and C35—H⋯*Cg*(C16–C21)^vi^ are labeled as 1–4, respectively. Two subconnectivities, which are orthogonal to the drawing plane, are marked with gray strips and they are detailed in the projections on the *ac* plane: (*b*) phenyl­sulfonyl layer; (*c*) π–π and C–H⋯O inter­actions between the mol­ecules of **II**. [Symmetry codes: (i) *x* − 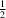, −*y* + 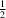, *z* − 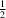; (ii) *x* + 1, *y*, *z*; (iii) *x* + 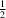, −*y* + 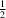, *z* − 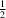; (iv) −*x* + 1, −*y* + 1, −*z* + 1; (vi) *x* − 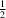, −*y* − 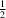, *z* − 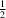; (vii) *x* − 1, *y*, *z* − 1.]

One can note that in **I**, and also in other comparable 1-(phenyl­sulfon­yl)-1*H*-indoles (Madhan *et al.*, 2024*a* ▸), the favorable π–π bonded duo is generated due to the inter­actions at only one axial side of the indole ring system. Therefore, in the case of **II**, the inter­molecular π–π inter­actions of the latter are completely suppressed due to the generation of the intra­molecular indole–indole stack. This is in line with the increased significance of C—H⋯O inter­actions in the crystal of **II**. The shortest hydrogen-bond contacts are observed for sulfonic O-atom acceptors [C27⋯O1^iv^ = 3.389 (6) Å; symmetry code: (iv) −*x* + 1, −*y* + 1, −*z* + 1]. These bonds assemble pairs of the mol­ecules into centrosymmetric dimers (Fig. 4 ▸) with a cyclic 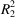
(13) (Bernstein *et al.*, 1995 ▸) ring motif. The dimers are further integrated into a three-dimensional framework. The amino-bound phenyl­sulfonyl groups are held together by a set of C—H⋯O bonds [*viz*. C18⋯O3^ii^ = 3.575 (6) and C19⋯O4^iii^ = 3.443 (6) Å; symmetry codes: (ii) *x* + 1, *y*, *z*; (iii) *x* + 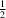, −*y* + 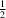, *z* − 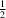] and constitute their own layer connectivities in the form of flat square nets, which are parallel to the *ac* plane (Fig. 4 ▸*b*). These layers are separated by 17.39 Å, which is half of the *b*-axis parameter of the unit cell, and are linked *via* bis­(indole­meth­yl)amine fragments of the above dimers (Fig. 4 ▸*b*). These bis­(indole­meth­yl)amine fragments themselves afford nominal layers with a set of weak inter­molecular inter­actions, such as C—H⋯O bonds [C34⋯O5^ii^ = 3.360 (6) Å; symmetry code: (ii) *x* + 1, *y*, *z*] and relatively distal π–π inter­actions between the outer phenyl rings, with an inter­centroid distance of 3.952 (3) Å (Fig. 4 ▸*c*).

## 4. Hirshfeld surface analysis

**Figure 5:**
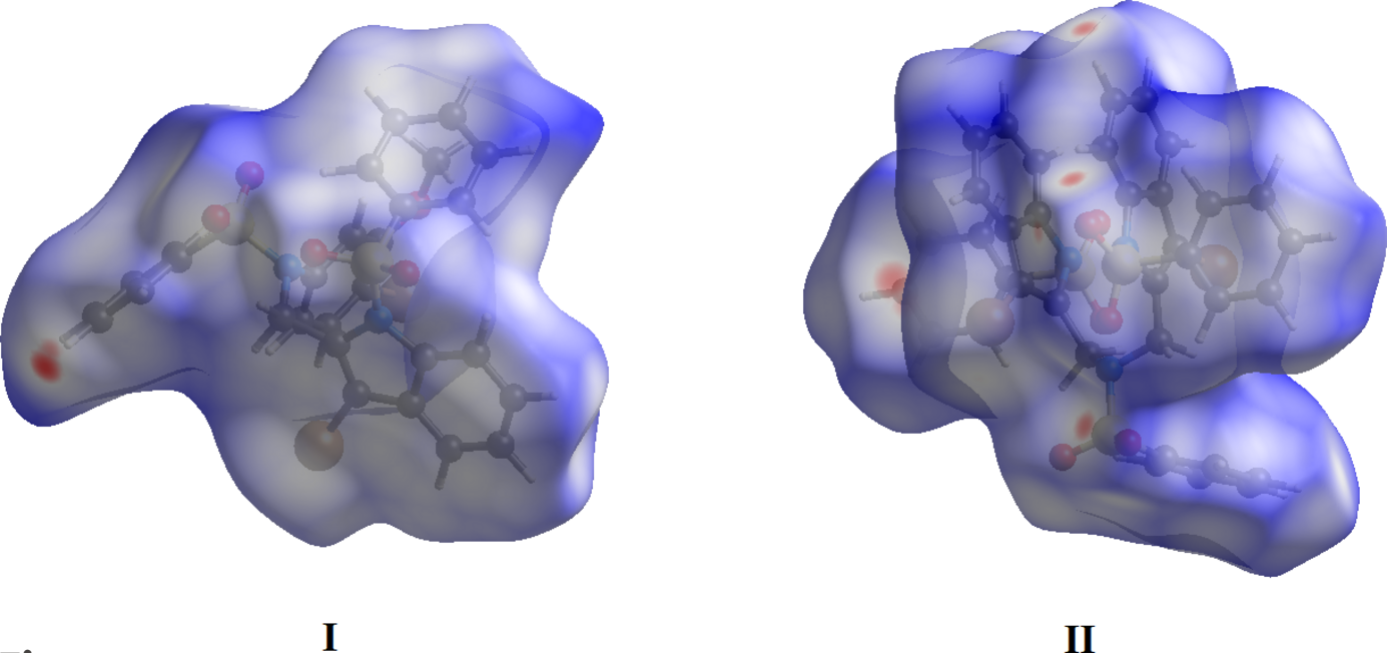
The Hirshfeld surfaces of compounds **I** and **II** mapped over *d_norm_*.

In order to investigate the weak inter­molecular inter­actions in the crystal, the Hirshfeld surfaces (*d*_norm_, curvedness and shape-index) and 2D fingerprint plots were generated using *Crystal Explorer 17.5* (Spackman *et al.*, 2021 ▸). The *d*_norm_ mapping uses the normalized functions of *d*_i_ and *d*_e_ (Fig. 5 ▸), with white surfaces indicating contacts with distances equal to the sum of van der Waals (vdW) radii, while red and blue colors reflect contacts at the distances below and above sum of the corresponding vdW radii, respectively.

**Figure 6:**
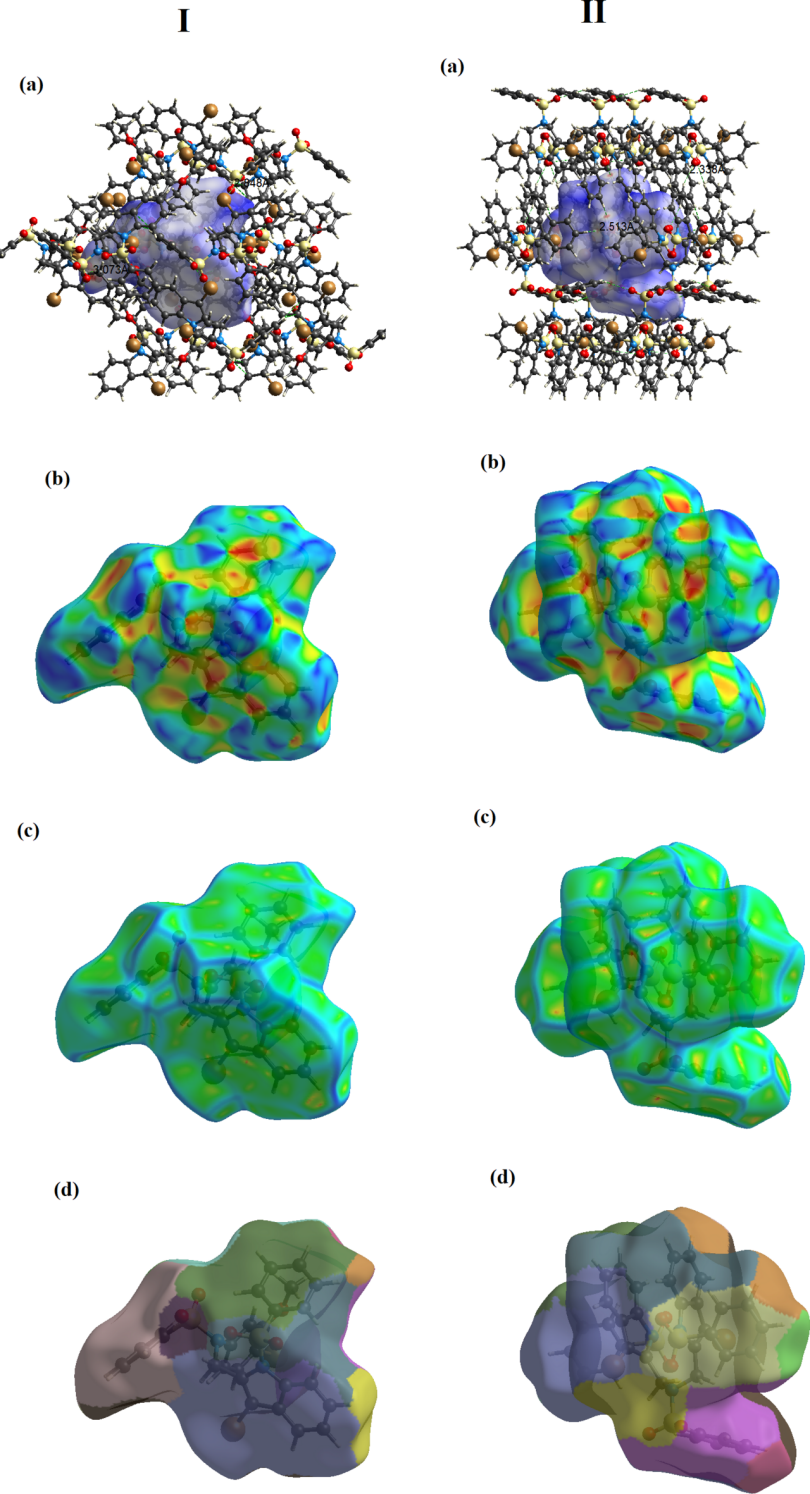
Hirshfeld surfaces for visualizing the inter­molecular contacts of the title compounds: (*a*) electrostatic potential, (*b*) shape-index, (*c*) curvedness and (*d*) fragment patches.

The Hirshfeld surfaces for two compounds mapped over *d*_norm_ using a fixed color scale of −0.125 (red) to 1.678 a.u. (blue) for **I** and −0.198 (red) to 1.491 a.u. (blue) for **II** are shown in Fig. 5 ▸. One can note weakness of inter­molecular bonding in a system that is, particularly the case of **I**, showing preferably normal van der Waals separations (denoted with several white regions on the surface). The only identified pair of diffuse red spots corresponds to C—H⋯O bonds. In the case of **II**, the observed low intense and diffuse red spots are slightly larger in number, which supports the increased significance of weak hydrogen-bonding inter­actions. The electrostatic potential was also mapped on the Hirshfeld surface using a STO-3G basis set and the Hartree–Fock level of theory (Spackman & Jayatilaka, 2009 ▸). The C—H⋯O hydrogen-bond donors and acceptors are shown as blue and red regions around the atoms corresponding to positive and negative electrostatic potentials, respectively (Fig. 6 ▸*a*). The presence of π–π stacking inter­actions is indicated by red and blue triangles on the shape-index surface (Fig. 6 ▸*b*). Areas on the Hirshfeld surface with high curvedness tend to divide the surface into contact patches with each neighboring mol­ecule. The coordination number in the crystal is defined by the curvedness of the Hirshfeld surface (Fig. 6 ▸*c*). The nearest neighbor in the coordination environment of a mol­ecule is identified from the color patches on the Hirshfeld surface depending on their closeness to adjacent mol­ecules (Fig. 6 ▸*d*).

**Figure 7:**
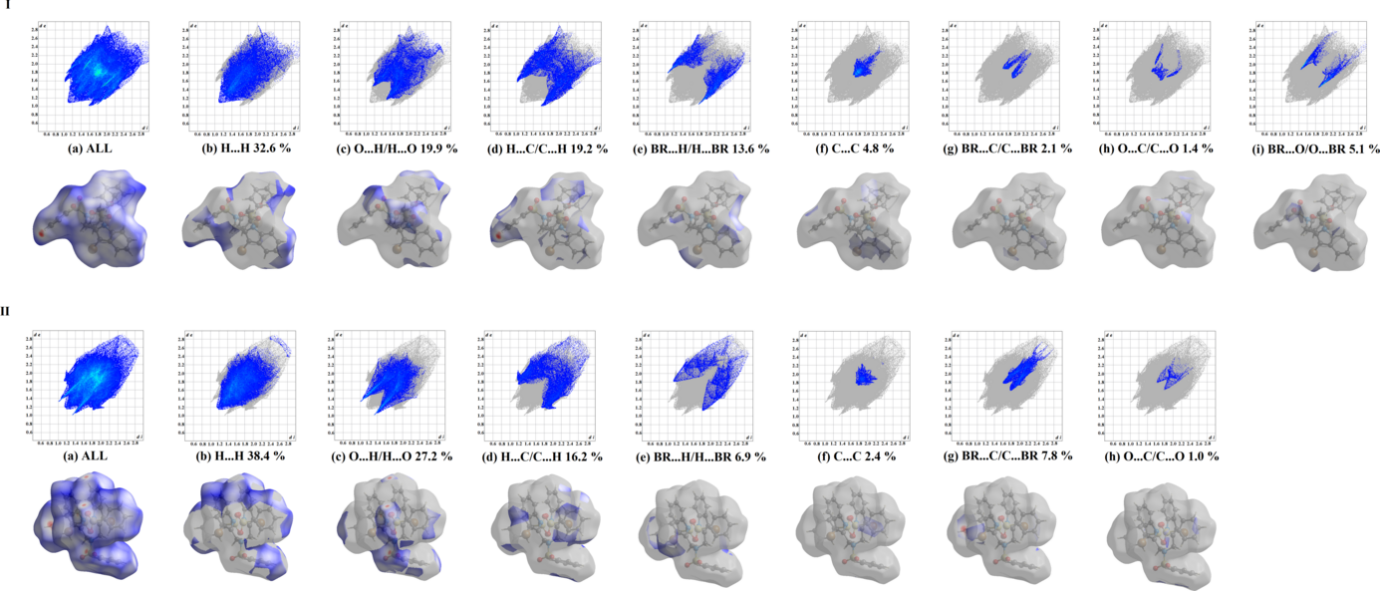
Two-dimensional fingerprint plots for **I** and **II** for all contacts and delineated into the principal contributions of H⋯H, O⋯H/H⋯O, C⋯H/H⋯C, Br.·H/H⋯Br, Br⋯O/O⋯Br, C⋯C, Br⋯C/C⋯Br and O⋯C/C⋯O contacts. Other contributors account for less than 1.0% of contacts to the surface areas.

Two-dimensional fingerprint plots showing the occurrence of all inter­molecular contacts (McKinnon *et al.*, 2007 ▸) are presented in Fig. 7 ▸. The plots for H⋯H contacts (Fig. 7 ▸*b*), which represent the largest contributions to the Hirshfeld surfaces (over 30%), show a distinct pattern with a minimum value of *d*_e_ = *d*_i_ = 1.1 Å. Beyond these largest fractions, the short contacts are overwhelmingly O⋯H/H⋯O (Fig. 7 ▸*c*) and C⋯H/H⋯C (Fig. 7 ▸*d*), which deliver as much as 19.9 and 19.2%, respectively, to the Hirshfeld surface in **I** and 27.2 and 16.2% in **II**. The significant increase in the O⋯H/H⋯O contributions when moving from **I** to **II** reflects the growing significance of C—H⋯O binding. This is in line with a larger number of the available O-atom acceptors in the latter case, but also it is a consequence of the elimination of inter­molecular π–π indole bonding. Accordingly, the pair of spikes identifying O⋯H/H⋯O contacts on the plots is more diffuse in the case of **I**. We note also a suppression of Br⋯H/H⋯Br contacts (6.9% for **II***versus* 13.6% for **I**). This fact does not provide a basis for comparison of the acceptor abilities of the indole- and phenyl-bound Br atoms, but rather reflects the steric unavailability of Br in **II** due to the forced intra­molecular inter­actions with sulfonyl O atoms. It is worth mentioning that the accumulation of unfavorable Br⋯O contacts within the mol­ecule of **II** causes the elimination of such contacts between the mol­ecules. This situation is evidenced by markedly different contributions of Br⋯O/O⋯Br contacts to the surface areas, which are 5.1% for **I**, but are completely absent in the case of **II**. An overlap between nearly parallel aromatic frames, due to the slipped π–π inter­actions, is clearly indicated by the C⋯C plots in the form of blue–green areas centered at *ca d*_e_ = *d*_i_ = 1.8 Å. A 50% decrease in the C⋯C contacts (2.4% for **II***versus* 4.8% for **I**) is also a consequence of intra­molecular indole–indole stacking, which mitigates against similar in nature inter­molecular inter­actions.

In brief, the Hirshfeld surface analysis confirms the importance of weak hydrogen bonding and contacts associated with the π–π inter­actions in establishing the packing. These results complement the main merit of the structure analysis and in total they suggest the possibility of controling the supra­molecular behavior of sulfonyl­ated indoles as possible biomedical materials.

## 5. Database survey

A search of the Cambridge Structural Database (Version 5.37; Groom *et al.*, 2016 ▸) indicated 123 compounds incorporating the phenyl­sulfonyl-1*H*-indole moiety. Of these, the most closely related examples are provided by structures of bromo­substituted 3-methyl-1-(phenyl­sulfon­yl)-1*H*-indole derivatives (JOMJII, JOMJAA and JOMJEE; Madhan *et al.*, 2024*b* ▸), ethyl 2-acet­oxy­methyl-1-phenyl­sulfonyl-1*H*-indole-3-carboxyl­ate (HUCQUS; Gunasekaran *et al.*, 2009 ▸), 3-iodo-2-methyl-1-phenyl­sulfonyl-1*H*-indole (ULESEK; Ramathilagam *et al.*, 2011 ▸) and 1-(2-bromo­methyl-1-phenyl­sulfonyl-1*H*-indol-3-yl) propan-1-one (CIQFEP; Umadevi *et al.*, 2013 ▸). In these structures, the sulfonyl-bound phenyl rings are almost orthogonal to the indole ring systems, with the corresponding dihedral angles lying in the range 73.35 (7)–89.91 (11)°.

## 6. Synthesis and crystallization

Compound **I**: To a solution of *N*-(3-meth­oxy­phen­yl)-*N*-{[1-(phenyl­sulfon­yl)-1*H*-indol-2-yl]meth­yl}benzene­sulfonamide (0.45 g, 0.845 mmol) in 5 ml of dry CH_2_Cl_2_, a mixture of phenyl­iodo­nium di­acetate (0.40 g, 1.268 mmol) and CuBr_2_ (0.56 g, 2.537 mmol) in 10 ml of CH_2_Cl_2_ was slowly added at 273 K. The reaction mixture was allowed to stir for 3 h at 273 K under an N_2_ atmosphere. After completion of the reaction (monitored by TLC), it was poured over cooled saturated aqueous NaHCO_3_ solution (20 mL) and then extracted with CH_2_Cl_2_ (2 × 10 mL). The extract was dried over Na_2_SO_4_. Removal of the solvent followed by recrystallization of the crude product from 5 mL of methanol afforded *N*-{[3-bromo-1-(phenyl­sulfon­yl)-1*H*-indol-2-yl]meth­yl}-*N*-(4-bromo-3-meth­oxy­phen­yl)benzene­sulfonamide (0.45 g, 78%) as a colorless solid, m.p. = 495–496 K. ^1^H NMR (300 MHz, CDCl_3_), δ, p.p.m.: 7.96 (*d*, *J* = 8.4 Hz, 1H), 7.69–7.64 (*m*, 4H), 7.57–7.34 (*m*, 6H), 7.29–7.24 (*m*, 2H), 7.20–7.11 (*m*, 2H), 6.36 (*s*, 1H), 6.25 (*d*, *J* = 8.4 Hz, 1H), 5.28 (*s*, 2H), 3.49 (*s*, 3H). ^13^C{^1^H} NMR (75 MHz, CDCl_3_), δ, p.p.m.: 155.4, 138.0, 137.9, 137.4, 136.3, 134.2, 133.1, 132.6, 130.2, 129.4, 128.8, 128.4, 128.2, 126.8, 126.6, 124.5, 122.7, 120.1, 115.2, 113.6, 111.7, 107.7, 56.1, 45.8. DEPT-135 ^13^C NMR (CDCl_3_), δ, p.p.m.: 134.2, 133.1, 132.6, 129.4, 128.9, 128.2, 126.8, 126.6, 124.5, 122.7, 120.2, 115.2, 113.6, 56.1, 45.8. HRMS (ESI) *m/z*: [*M*+H]^+^ Calculated for C_28_H_23_^79^Br_2_N_2_O_5_S_2_: 688.9415; found: 688.9407.

Compound **II**: To a solution of *N*,*N*-bis­{[1-(phenyl­sulfon­yl)-1*H*-indol-2-yl]meth­yl}benzene­sulfonamide (0.25 g, 0.359 mmol) in 5 ml of dry CH_2_Cl_2_, a mixture of phenyl­iodo­nium di­acetate (0.23 g, 0.719 mmol) and CuBr_2_ (0.24 g, 1.079 mmol) in 10 ml of CH_2_Cl_2_ was slowly added at 273 K. The reaction mixture was allowed to stir for 3 h at 273 K under an N_2_ atmosphere. After completion of the reaction (monitored by TLC), it was poured over cooled saturated aqueous NaHCO_3_ solution (20 mL) and then extracted with CH_2_Cl_2_ (2 × 10 mL). The extract was dried over Na_2_SO_4_. Removal of the solvent followed by recrystallization of the crude product from 5 ml of methanol afforded *N*,*N*-bis­{[3-bromo-1-(phenyl­sulfon­yl)-1*H*-indol-2-yl]meth­yl}benzene­sulfonamide (0.15 g, 60%) as a colorless solid, m.p. = 529–531 K. ^1^H NMR (300 MHz, CDCl_3_), δ, p.p.m.: 7.93 (*d*, *J* = 8.1 Hz, 2H), 7.77 (*d*, *J* = 7.5 Hz, 2H), 7.52–7.45 (*m*, 6H), 7.38–7.19 (*m*, 13H), 7.14–7.09 (*m*, 2H), 5.14 (*s*, 4H). ^13^C{^1^H} NMR (75 MHz, CDCl_3_), δ, p.p.m.: 138.9, 137.4, 136.6, 133.9, 131.9, 129.5, 129.2, 128.0, 127.5, 126.6, 126.3, 124.6, 120.0, 115.7, 109.2, 45.0.

## 7. Refinement

Crystal data, data collection and structure refinement details are summarized in Table 4 ▸. All hydrogen atoms were positioned geometrically and refined as riding with C—H = 0.93 Å (aromatic CH), 0.97 Å (CH_2_) and 0.96 Å (CH_3_) and 0.97 Å with *U*_iso_(H) = 1.5*U*_eq_(C) for methyl groups and 1.2U_eq_(C) for other H atoms.

## Supplementary Material

Crystal structure: contains datablock(s) global, I, II. DOI: 10.1107/S2056989024009587/nu2007sup1.cif

Structure factors: contains datablock(s) I. DOI: 10.1107/S2056989024009587/nu2007Isup2.hkl

Structure factors: contains datablock(s) II. DOI: 10.1107/S2056989024009587/nu2007IIsup3.hkl

Supporting information file. DOI: 10.1107/S2056989024009587/nu2007Isup4.cml

Supporting information file. DOI: 10.1107/S2056989024009587/nu2007IIsup5.cml

CCDC references: 2387808, 2350346

Additional supporting information:  crystallographic information; 3D view; checkCIF report

## Figures and Tables

**Table 1:** Hydrogen-bond geometry (Å, °) for **I**[Chem scheme1]

*D*—H⋯*A*	*D*—H	H⋯*A*	*D*⋯*A*	*D*—H⋯*A*
C2—H2⋯O1	0.93	2.39	2.908 (5)	115
C11—H11⋯O4^i^	0.93	2.76	3.503 (6)	137
C15—H15*B*⋯O2	0.97	2.31	2.886 (4)	117
C18—H18⋯*Cg*(N1/C1/C6–C8)^ii^	0.93	2.99	3.861 (8)	156
C18—H18⋯*Cg*(C1–C6)^ii^	0.93	2.81	3.579 (1)	141

Symmetry codes: (i) 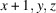; (ii) 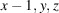.

**Table 2:** Hydrogen-bond geometry (Å, °) for **II**[Chem scheme1]

*D*—H⋯*A*	*D*—H	H⋯*A*	*D*⋯*A*	*D*—H⋯*A*
C2—H2⋯O1	0.93	2.43	2.993 (7)	119
C13—H13⋯O3^i^	0.93	2.80	3.324 (6)	117
C14—H14⋯O4^i^	0.93	2.78	3.558 (5)	142
C15—H15*B*⋯O2	0.97	2.25	2.850 (5)	119
C18—H18⋯O3^ii^	0.93	2.73	3.575 (6)	151
C19—H19⋯O4^iii^	0.93	2.59	3.443 (6)	152
C19—H19⋯O6^iii^	0.93	2.81	3.467 (6)	129
C20—H20⋯Br2^iii^	0.93	3.02	3.536 (5)	117
C22—H22*A*⋯O6	0.97	2.39	2.950 (5)	116
C27—H27⋯O1^iv^	0.93	2.48	3.389 (6)	164
C28—H28⋯O5^v^	0.93	2.63	3.547 (6)	170
C29—H29⋯O5	0.93	2.35	2.907 (5)	118
C34—H34⋯O5^ii^	0.93	2.62	3.360 (6)	137
C35—H35⋯*Cg*(C16–C21)^vi^	0.93	2.91	3.729 (7)	147

Symmetry codes: (i) 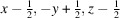; (ii) 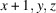; (iii) 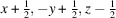; (iv) 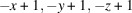; (v) 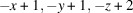; (vi) 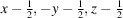.

**Table 3:** Geometry of stacking inter­actions (Å, °) for **I** and **II** *Cg* is a group centroid; plane⋯*CgB* is the distance between the mean plane of group *A* and the centroid of inter­acting group *B*; ipa is the inter­planar angle; sa is the slippage angle, which is the angle of the *CgA*⋯*CgB* axis to the group *A* mean plane normal.

Compound	Group *A*	Group *B*	Shortest contact	*CgA*⋯*CgB*	Plane⋯*CgB*	ipa	sa
**I**	(N1/C1/C6–C8)	(C1–C6)^iii^	3.456 (2)	3.532 (2)	3.450 (2)	1.2 (2)	12.4 (2)
	(C9–C14)	(C9–C14)^iv^	3.397 (2)	3.824 (2)	3.397 (2)	0	27.3 (2)
**II**	(N1/C1/C6–C8)	(N3/C30/C23–C25)	3.225 (2)	3.267 (2)	3.256 (2)	10.4 (3)	4.8 (2)
	(C1–C6)	(C25–C30)	3.499 (2)	3.593 (3)	3.531 (2)	4.9 (3)	10.7 (2)
	(C9–C14)	(C31–C36)^vii^	3.464 (2)	3.952 (3)	3.636 (2)	6.64 (16)	23.0 (2)

Symmetry codes for **I**: (iii) −*x* + 1, −*y* + 1, −*z* + 1; (iv) −*x* + 1, −*y* + 2, −*z* + 1; for **II**: (vii) *x* − 1, *y*, *z* − 1.

**Table 4:** Experimental details

	**I**	**II**
Crystal data		
Chemical formula	C_28_H_22_Br_2_N_2_O_5_S_2_	C_36_H_27_Br_2_N_3_O_6_S_3_
*M*_r_	690.41	853.60
Crystal system, space group	Monoclinic, *P*2_1_/*n*	Monoclinic, *P*2_1_/*n*
Temperature (K)	303	303
*a*, *b*, *c* (Å)	9.5718 (6), 14.4498 (8), 20.0041 (12)	8.2664 (4), 34.7886 (18), 12.5972 (6)
β (°)	92.874 (2)	104.550 (2)
*V* (Å^3^)	2763.3 (3)	3506.5 (3)
*Z*	4	4
Radiation type	Mo *K*α	Mo *K*α
μ (mm^−1^)	3.13	2.54
Crystal size (mm)	0.29 × 0.24 × 0.20	0.29 × 0.19 × 0.04

Data collection		
Diffractometer	Bruker D8 Venture Diffractometer	Bruker D8 Venture Diffractometer
Absorption correction	Multi-scan (*SADABS*; Krause *et al.*, 2015 ▸)	Multi-scan (*SADABS*; Krause *et al.*, 2015 ▸)
*T*_min_, *T*_max_	0.589, 0.753	0.491, 0.745
No. of measured, independent and observed [*I* > 2σ(*I*)] reflections	90697, 5234, 4173	73921, 6434, 5196
*R*_int_	0.071	0.071

Refinement		
*R*[*F*^2^ > 2σ(*F*^2^)], *wR*(*F*^2^), *S*	0.040, 0.099, 1.05	0.048, 0.120, 1.12
No. of reflections	5234	6434
No. of parameters	353	451
H-atom treatment	H-atom parameters constrained	H-atom parameters constrained
Δρ_max_, Δρ_min_ (e Å^−3^)	1.74, −1.41	0.46, −0.47

Computer programs: *APEX2* and *SAINT* (Bruker, 2016 ▸), *SHELXT2018/2* (Sheldrick, 2015*a* ▸), *SHELXL2019/2* (Sheldrick, 2015*b* ▸), *ORTEP-3 for Windows* (Farrugia, 2012 ▸) and *Mercury* (Macrae *et al.*, 2020 ▸), *WinGX* (Farrugia, 2012 ▸), *publCIF* (Westrip, 2010 ▸) and *PLATON* (Spek, 2020 ▸).

## supplementary crystallographic information

### N-{[3-Bromo-1-(phenylsulfonyl)-1H-indol-2-yl]methyl}-N-(4-bromo-3-methoxyphenyl)benzenesulfonamide (I) Crystal data

**Table:**

C_28_H_22_Br_2_N_2_O_5_S_2_	*F*(000) = 1384
*M**_r_* = 690.41	*D*_x_ = 1.660 Mg m^−^^3^
Monoclinic, *P*2_1_/*n*	Mo *K*α radiation, λ = 0.71073 Å
*a* = 9.5718 (6) Å	Cell parameters from 90697 reflections
*b* = 14.4498 (8) Å	θ = 1.4–25.0°
*c* = 20.0041 (12) Å	µ = 3.13 mm^−^^1^
β = 92.874 (2)°	*T* = 303 K
*V* = 2763.3 (3) Å^3^	Block, colorless
*Z* = 4	0.29 × 0.24 × 0.20 mm

### N-{[3-Bromo-1-(phenylsulfonyl)-1H-indol-2-yl]methyl}-N-(4-bromo-3-methoxyphenyl)benzenesulfonamide (I) Data collection

**Table:**

Bruker D8 Venture Diffractometer	4173 reflections with *I* > 2σ(*I*)
Radiation source: micro focus sealed tube	*R*_int_ = 0.071
ω and φ scans	θ_max_ = 25.7°, θ_min_ = 2.5°
Absorption correction: multi-scan (*SADABS*; Krause *et al.*, 2015)	*h* = −11→11
*T*_min_ = 0.589, *T*_max_ = 0.753	*k* = −17→17
90697 measured reflections	*l* = −24→24
5234 independent reflections	

### N-{[3-Bromo-1-(phenylsulfonyl)-1H-indol-2-yl]methyl}-N-(4-bromo-3-methoxyphenyl)benzenesulfonamide (I) Refinement

**Table:**

Refinement on *F*^2^	0 restraints
Least-squares matrix: full	Hydrogen site location: inferred from neighbouring sites
*R*[*F*^2^ > 2σ(*F*^2^)] = 0.040	H-atom parameters constrained
*wR*(*F*^2^) = 0.099	*w* = 1/[σ^2^(*F*_o_^2^) + (0.0293*P*)^2^ + 5.9561*P*] where *P* = (*F*_o_^2^ + 2*F*_c_^2^)/3
*S* = 1.05	(Δ/σ)_max_ = 0.001
5234 reflections	Δρ_max_ = 1.74 e Å^−^^3^
353 parameters	Δρ_min_ = −1.41 e Å^−^^3^

### N-{[3-Bromo-1-(phenylsulfonyl)-1H-indol-2-yl]methyl}-N-(4-bromo-3-methoxyphenyl)benzenesulfonamide (I) Special details

**Table:**

Geometry. All esds (except the esd in the dihedral angle between two l.s. planes) are estimated using the full covariance matrix. The cell esds are taken into account individually in the estimation of esds in distances, angles and torsion angles; correlations between esds in cell parameters are only used when they are defined by crystal symmetry. An approximate (isotropic) treatment of cell esds is used for estimating esds involving l.s. planes.

### N-{[3-Bromo-1-(phenylsulfonyl)-1H-indol-2-yl]methyl}-N-(4-bromo-3-methoxyphenyl)benzenesulfonamide (I) Fractional atomic coordinates and isotropic or equivalent isotropic displacement parameters (Å^2^)

**Table:**

	*x*	*y*	*z*	*U*_iso_*/*U*_eq_	
C1	0.5912 (4)	0.6131 (2)	0.46145 (17)	0.0360 (8)	
C2	0.7216 (4)	0.6188 (3)	0.4946 (2)	0.0485 (9)	
H2	0.746482	0.669420	0.521272	0.058*	
C3	0.8133 (5)	0.5462 (3)	0.4864 (2)	0.0581 (11)	
H3	0.901819	0.548866	0.507653	0.070*	
C4	0.7775 (5)	0.4695 (3)	0.4473 (2)	0.0575 (11)	
H4	0.841931	0.421970	0.443087	0.069*	
C5	0.6482 (5)	0.4633 (3)	0.41506 (19)	0.0486 (10)	
H5	0.623774	0.411785	0.389142	0.058*	
C6	0.5536 (4)	0.5362 (2)	0.42199 (17)	0.0372 (8)	
C7	0.4158 (4)	0.5529 (2)	0.39470 (17)	0.0397 (8)	
C8	0.3685 (4)	0.6356 (2)	0.41568 (16)	0.0337 (7)	
C9	0.5386 (4)	0.8611 (2)	0.46312 (18)	0.0401 (8)	
C10	0.6823 (5)	0.8709 (3)	0.4644 (3)	0.0610 (12)	
H10	0.740032	0.827279	0.485932	0.073*	
C11	0.7389 (6)	0.9465 (3)	0.4332 (3)	0.0775 (16)	
H11	0.835512	0.953850	0.433711	0.093*	
C12	0.6537 (6)	1.0105 (3)	0.4017 (2)	0.0686 (14)	
H12	0.692625	1.061212	0.380876	0.082*	
C13	0.5117 (6)	1.0007 (3)	0.4006 (2)	0.0617 (12)	
H13	0.454818	1.044675	0.379011	0.074*	
C14	0.4518 (5)	0.9258 (3)	0.43145 (19)	0.0479 (9)	
H14	0.355072	0.919091	0.430866	0.057*	
C15	0.2288 (4)	0.6783 (3)	0.39902 (17)	0.0374 (8)	
H15A	0.168446	0.632698	0.376678	0.045*	
H15B	0.186408	0.696104	0.440144	0.045*	
C16	−0.0367 (4)	0.7764 (3)	0.31156 (18)	0.0404 (8)	
C17	−0.1273 (4)	0.7228 (3)	0.3466 (2)	0.0544 (11)	
H17	−0.117432	0.718441	0.392956	0.065*	
C18	−0.2334 (5)	0.6754 (3)	0.3113 (2)	0.0640 (12)	
H18	−0.296109	0.639804	0.334362	0.077*	
C19	−0.2469 (4)	0.6806 (3)	0.2427 (2)	0.0564 (11)	
H19	−0.317930	0.648112	0.219529	0.068*	
C20	−0.1556 (4)	0.7338 (3)	0.2082 (2)	0.0557 (11)	
H20	−0.165121	0.737115	0.161734	0.067*	
C21	−0.0501 (4)	0.7820 (3)	0.2420 (2)	0.0508 (10)	
H21	0.011626	0.818004	0.218646	0.061*	
C22	0.2982 (4)	0.7476 (2)	0.29018 (16)	0.0347 (8)	
C23	0.2612 (4)	0.6733 (3)	0.24960 (19)	0.0453 (9)	
H23	0.195602	0.630544	0.263040	0.054*	
C24	0.3227 (4)	0.6627 (3)	0.18851 (18)	0.0482 (10)	
H24	0.300825	0.611588	0.161749	0.058*	
C25	0.4159 (4)	0.7279 (3)	0.16766 (17)	0.0395 (8)	
C26	0.4523 (4)	0.8037 (3)	0.20774 (18)	0.0405 (8)	
C27	0.3937 (4)	0.8122 (3)	0.26980 (18)	0.0386 (8)	
H27	0.418840	0.861639	0.297649	0.046*	
C28	0.5954 (7)	0.9366 (4)	0.2262 (3)	0.090 (2)	
H28A	0.519832	0.977821	0.235067	0.135*	
H28B	0.667708	0.970260	0.205184	0.135*	
H28C	0.632434	0.910622	0.267595	0.135*	
N1	0.4763 (3)	0.67482 (19)	0.45867 (14)	0.0344 (6)	
N2	0.2386 (3)	0.7612 (2)	0.35510 (14)	0.0356 (6)	
O1	0.5542 (3)	0.75110 (19)	0.56538 (13)	0.0516 (7)	
O2	0.3206 (3)	0.78625 (18)	0.51436 (13)	0.0488 (7)	
O3	0.1411 (3)	0.91308 (19)	0.31900 (14)	0.0516 (7)	
O4	0.0678 (3)	0.8422 (2)	0.42399 (13)	0.0552 (7)	
O5	0.5454 (3)	0.8642 (2)	0.18304 (14)	0.0589 (8)	
S1	0.46528 (10)	0.76919 (6)	0.50760 (4)	0.0375 (2)	
S2	0.10295 (10)	0.83326 (7)	0.35581 (5)	0.0412 (2)	
Br1	0.50238 (5)	0.71099 (4)	0.08522 (2)	0.05801 (14)	
Br2	0.31588 (6)	0.46768 (3)	0.34009 (2)	0.06813 (17)	

### N-{[3-Bromo-1-(phenylsulfonyl)-1H-indol-2-yl]methyl}-N-(4-bromo-3-methoxyphenyl)benzenesulfonamide (I) Atomic displacement parameters (Å^2^)

**Table:**

	*U*^11^	*U*^22^	*U*^33^	*U*^12^	*U*^13^	*U*^23^
C1	0.0391 (19)	0.0333 (18)	0.0364 (18)	0.0038 (15)	0.0097 (15)	0.0063 (15)
C2	0.043 (2)	0.044 (2)	0.059 (2)	0.0010 (17)	0.0015 (18)	0.0019 (19)
C3	0.044 (2)	0.062 (3)	0.068 (3)	0.013 (2)	0.003 (2)	0.009 (2)
C4	0.060 (3)	0.052 (3)	0.061 (3)	0.024 (2)	0.014 (2)	0.009 (2)
C5	0.067 (3)	0.038 (2)	0.042 (2)	0.0142 (19)	0.0155 (19)	0.0004 (17)
C6	0.048 (2)	0.0349 (18)	0.0295 (17)	0.0050 (16)	0.0095 (15)	0.0036 (14)
C7	0.054 (2)	0.0360 (19)	0.0289 (17)	0.0034 (17)	0.0023 (16)	−0.0022 (15)
C8	0.042 (2)	0.0340 (18)	0.0251 (16)	−0.0003 (15)	0.0038 (14)	0.0010 (14)
C9	0.050 (2)	0.0294 (18)	0.042 (2)	0.0005 (16)	0.0067 (16)	−0.0062 (15)
C10	0.049 (3)	0.038 (2)	0.097 (4)	0.0055 (19)	0.016 (2)	0.003 (2)
C11	0.066 (3)	0.054 (3)	0.117 (5)	−0.004 (2)	0.041 (3)	−0.002 (3)
C12	0.102 (4)	0.038 (2)	0.068 (3)	−0.008 (3)	0.032 (3)	−0.001 (2)
C13	0.096 (4)	0.038 (2)	0.051 (3)	0.004 (2)	−0.003 (2)	−0.0018 (19)
C14	0.058 (2)	0.039 (2)	0.045 (2)	0.0029 (18)	−0.0065 (18)	−0.0071 (17)
C15	0.0388 (19)	0.042 (2)	0.0314 (17)	−0.0015 (16)	0.0040 (14)	0.0008 (15)
C16	0.0337 (19)	0.045 (2)	0.043 (2)	0.0061 (16)	0.0080 (15)	0.0088 (16)
C17	0.051 (2)	0.070 (3)	0.044 (2)	−0.001 (2)	0.0175 (19)	0.011 (2)
C18	0.054 (3)	0.071 (3)	0.069 (3)	−0.010 (2)	0.025 (2)	0.011 (2)
C19	0.041 (2)	0.059 (3)	0.069 (3)	0.000 (2)	0.000 (2)	0.004 (2)
C20	0.053 (3)	0.071 (3)	0.043 (2)	−0.002 (2)	−0.0014 (19)	0.011 (2)
C21	0.046 (2)	0.063 (3)	0.044 (2)	−0.006 (2)	0.0044 (18)	0.0190 (19)
C22	0.0319 (18)	0.043 (2)	0.0296 (17)	0.0069 (15)	0.0018 (14)	0.0011 (15)
C23	0.044 (2)	0.053 (2)	0.0390 (19)	−0.0077 (18)	0.0043 (16)	−0.0029 (17)
C24	0.050 (2)	0.061 (3)	0.0338 (19)	−0.004 (2)	−0.0002 (17)	−0.0115 (18)
C25	0.0339 (19)	0.057 (2)	0.0271 (16)	0.0076 (17)	0.0033 (14)	−0.0004 (16)
C26	0.0366 (19)	0.045 (2)	0.0407 (19)	0.0022 (16)	0.0082 (15)	0.0017 (17)
C27	0.0382 (19)	0.0396 (19)	0.0384 (19)	0.0015 (16)	0.0060 (15)	−0.0028 (15)
C28	0.109 (4)	0.078 (4)	0.086 (4)	−0.049 (3)	0.052 (3)	−0.028 (3)
N1	0.0377 (16)	0.0299 (14)	0.0358 (15)	0.0025 (12)	0.0041 (12)	−0.0035 (12)
N2	0.0369 (16)	0.0411 (16)	0.0291 (14)	0.0056 (13)	0.0047 (12)	0.0027 (12)
O1	0.0678 (19)	0.0501 (16)	0.0359 (14)	0.0026 (14)	−0.0065 (13)	−0.0033 (12)
O2	0.0461 (15)	0.0508 (16)	0.0508 (16)	0.0035 (13)	0.0147 (12)	−0.0137 (13)
O3	0.0532 (17)	0.0416 (15)	0.0599 (17)	0.0042 (13)	0.0024 (13)	0.0061 (13)
O4	0.0559 (17)	0.0688 (19)	0.0412 (15)	0.0214 (15)	0.0073 (13)	−0.0074 (14)
O5	0.068 (2)	0.0602 (18)	0.0507 (17)	−0.0152 (15)	0.0275 (15)	−0.0064 (14)
S1	0.0441 (5)	0.0353 (4)	0.0334 (4)	0.0024 (4)	0.0055 (4)	−0.0053 (4)
S2	0.0402 (5)	0.0451 (5)	0.0384 (5)	0.0088 (4)	0.0055 (4)	0.0014 (4)
Br1	0.0547 (3)	0.0844 (3)	0.0358 (2)	0.0026 (2)	0.01070 (17)	−0.0083 (2)
Br2	0.0946 (4)	0.0496 (3)	0.0575 (3)	0.0103 (2)	−0.0228 (2)	−0.0210 (2)

### N-{[3-Bromo-1-(phenylsulfonyl)-1H-indol-2-yl]methyl}-N-(4-bromo-3-methoxyphenyl)benzenesulfonamide (I) Geometric parameters (Å, º)

**Table:**

C1—C2	1.386 (5)	C16—S2	1.768 (4)
C1—C6	1.399 (5)	C17—C18	1.388 (6)
C1—N1	1.415 (4)	C17—H17	0.9300
C2—C3	1.382 (6)	C18—C19	1.375 (6)
C2—H2	0.9300	C18—H18	0.9300
C3—C4	1.388 (6)	C19—C20	1.375 (6)
C3—H3	0.9300	C19—H19	0.9300
C4—C5	1.370 (6)	C20—C21	1.377 (6)
C4—H4	0.9300	C20—H20	0.9300
C5—C6	1.400 (5)	C21—H21	0.9300
C5—H5	0.9300	C22—C23	1.381 (5)
C6—C7	1.423 (5)	C22—C27	1.383 (5)
C7—C8	1.352 (5)	C22—N2	1.457 (4)
C7—Br2	1.876 (4)	C23—C24	1.391 (5)
C8—N1	1.427 (4)	C23—H23	0.9300
C8—C15	1.495 (5)	C24—C25	1.377 (5)
C9—C10	1.381 (6)	C24—H24	0.9300
C9—C14	1.384 (5)	C25—C26	1.391 (5)
C9—S1	1.764 (4)	C25—Br1	1.898 (3)
C10—C11	1.382 (6)	C26—O5	1.358 (4)
C10—H10	0.9300	C26—C27	1.394 (5)
C11—C12	1.367 (7)	C27—H27	0.9300
C11—H11	0.9300	C28—O5	1.425 (6)
C12—C13	1.365 (7)	C28—H28A	0.9600
C12—H12	0.9300	C28—H28B	0.9600
C13—C14	1.384 (6)	C28—H28C	0.9600
C13—H13	0.9300	N1—S1	1.685 (3)
C14—H14	0.9300	N2—S2	1.665 (3)
C15—N2	1.491 (4)	O1—S1	1.425 (3)
C15—H15A	0.9700	O2—S1	1.420 (3)
C15—H15B	0.9700	O3—S2	1.426 (3)
C16—C17	1.380 (5)	O4—S2	1.427 (3)
C16—C21	1.393 (5)		
			
C2—C1—C6	121.1 (3)	C19—C18—H18	119.7
C2—C1—N1	131.4 (3)	C17—C18—H18	119.7
C6—C1—N1	107.5 (3)	C18—C19—C20	120.1 (4)
C3—C2—C1	117.4 (4)	C18—C19—H19	120.0
C3—C2—H2	121.3	C20—C19—H19	120.0
C1—C2—H2	121.3	C19—C20—C21	120.4 (4)
C2—C3—C4	122.2 (4)	C19—C20—H20	119.8
C2—C3—H3	118.9	C21—C20—H20	119.8
C4—C3—H3	118.9	C20—C21—C16	119.3 (4)
C5—C4—C3	120.6 (4)	C20—C21—H21	120.3
C5—C4—H4	119.7	C16—C21—H21	120.3
C3—C4—H4	119.7	C23—C22—C27	120.4 (3)
C4—C5—C6	118.5 (4)	C23—C22—N2	121.9 (3)
C4—C5—H5	120.8	C27—C22—N2	117.7 (3)
C6—C5—H5	120.8	C22—C23—C24	119.6 (4)
C1—C6—C5	120.3 (4)	C22—C23—H23	120.2
C1—C6—C7	106.8 (3)	C24—C23—H23	120.2
C5—C6—C7	132.9 (4)	C25—C24—C23	120.0 (4)
C8—C7—C6	110.5 (3)	C25—C24—H24	120.0
C8—C7—Br2	126.3 (3)	C23—C24—H24	120.0
C6—C7—Br2	123.2 (3)	C24—C25—C26	120.8 (3)
C7—C8—N1	107.1 (3)	C24—C25—Br1	119.5 (3)
C7—C8—C15	127.2 (3)	C26—C25—Br1	119.6 (3)
N1—C8—C15	125.6 (3)	O5—C26—C25	116.5 (3)
C10—C9—C14	120.9 (4)	O5—C26—C27	124.6 (3)
C10—C9—S1	119.3 (3)	C25—C26—C27	118.8 (3)
C14—C9—S1	119.6 (3)	C22—C27—C26	120.3 (3)
C9—C10—C11	119.1 (4)	C22—C27—H27	119.8
C9—C10—H10	120.5	C26—C27—H27	119.8
C11—C10—H10	120.5	O5—C28—H28A	109.5
C12—C11—C10	120.3 (5)	O5—C28—H28B	109.5
C12—C11—H11	119.9	H28A—C28—H28B	109.5
C10—C11—H11	119.9	O5—C28—H28C	109.5
C13—C12—C11	120.5 (4)	H28A—C28—H28C	109.5
C13—C12—H12	119.8	H28B—C28—H28C	109.5
C11—C12—H12	119.8	C1—N1—C8	108.1 (3)
C12—C13—C14	120.6 (4)	C1—N1—S1	124.0 (2)
C12—C13—H13	119.7	C8—N1—S1	127.4 (2)
C14—C13—H13	119.7	C22—N2—C15	117.1 (3)
C9—C14—C13	118.6 (4)	C22—N2—S2	115.6 (2)
C9—C14—H14	120.7	C15—N2—S2	115.1 (2)
C13—C14—H14	120.7	C26—O5—C28	117.3 (3)
N2—C15—C8	112.3 (3)	O2—S1—O1	120.03 (17)
N2—C15—H15A	109.1	O2—S1—N1	106.60 (15)
C8—C15—H15A	109.1	O1—S1—N1	105.69 (15)
N2—C15—H15B	109.1	O2—S1—C9	109.41 (18)
C8—C15—H15B	109.1	O1—S1—C9	108.10 (18)
H15A—C15—H15B	107.9	N1—S1—C9	106.14 (15)
C17—C16—C21	120.7 (4)	O3—S2—O4	119.92 (18)
C17—C16—S2	119.0 (3)	O3—S2—N2	106.35 (16)
C21—C16—S2	120.1 (3)	O4—S2—N2	106.66 (15)
C16—C17—C18	118.8 (4)	O3—S2—C16	108.91 (17)
C16—C17—H17	120.6	O4—S2—C16	108.18 (18)
C18—C17—H17	120.6	N2—S2—C16	105.98 (16)
C19—C18—C17	120.7 (4)		
			
C6—C1—C2—C3	0.8 (6)	Br1—C25—C26—C27	−176.4 (3)
N1—C1—C2—C3	−179.1 (4)	C23—C22—C27—C26	0.8 (5)
C1—C2—C3—C4	−0.8 (6)	N2—C22—C27—C26	−178.9 (3)
C2—C3—C4—C5	0.2 (7)	O5—C26—C27—C22	179.1 (3)
C3—C4—C5—C6	0.4 (6)	C25—C26—C27—C22	−1.7 (5)
C2—C1—C6—C5	−0.2 (5)	C2—C1—N1—C8	178.6 (4)
N1—C1—C6—C5	179.7 (3)	C6—C1—N1—C8	−1.2 (4)
C2—C1—C6—C7	−179.1 (3)	C2—C1—N1—S1	−9.6 (5)
N1—C1—C6—C7	0.8 (4)	C6—C1—N1—S1	170.5 (2)
C4—C5—C6—C1	−0.4 (5)	C7—C8—N1—C1	1.2 (4)
C4—C5—C6—C7	178.1 (4)	C15—C8—N1—C1	179.8 (3)
C1—C6—C7—C8	−0.1 (4)	C7—C8—N1—S1	−170.2 (3)
C5—C6—C7—C8	−178.7 (4)	C15—C8—N1—S1	8.4 (5)
C1—C6—C7—Br2	−177.9 (2)	C23—C22—N2—C15	44.3 (5)
C5—C6—C7—Br2	3.4 (6)	C27—C22—N2—C15	−136.0 (3)
C6—C7—C8—N1	−0.7 (4)	C23—C22—N2—S2	−96.5 (4)
Br2—C7—C8—N1	177.1 (2)	C27—C22—N2—S2	83.3 (3)
C6—C7—C8—C15	−179.3 (3)	C8—C15—N2—C22	59.7 (4)
Br2—C7—C8—C15	−1.5 (5)	C8—C15—N2—S2	−159.4 (2)
C14—C9—C10—C11	−0.3 (7)	C25—C26—O5—C28	−173.4 (4)
S1—C9—C10—C11	−175.7 (4)	C27—C26—O5—C28	5.8 (6)
C9—C10—C11—C12	0.1 (8)	C1—N1—S1—O2	−152.6 (3)
C10—C11—C12—C13	0.0 (8)	C8—N1—S1—O2	17.5 (3)
C11—C12—C13—C14	0.1 (7)	C1—N1—S1—O1	−23.9 (3)
C10—C9—C14—C13	0.3 (6)	C8—N1—S1—O1	146.3 (3)
S1—C9—C14—C13	175.7 (3)	C1—N1—S1—C9	90.8 (3)
C12—C13—C14—C9	−0.2 (6)	C8—N1—S1—C9	−99.1 (3)
C7—C8—C15—N2	−111.1 (4)	C10—C9—S1—O2	164.5 (3)
N1—C8—C15—N2	70.5 (4)	C14—C9—S1—O2	−11.0 (3)
C21—C16—C17—C18	−0.9 (6)	C10—C9—S1—O1	32.2 (4)
S2—C16—C17—C18	−177.5 (3)	C14—C9—S1—O1	−143.3 (3)
C16—C17—C18—C19	1.0 (7)	C10—C9—S1—N1	−80.8 (4)
C17—C18—C19—C20	−0.6 (7)	C14—C9—S1—N1	103.7 (3)
C18—C19—C20—C21	0.1 (7)	C22—N2—S2—O3	−46.6 (3)
C19—C20—C21—C16	0.1 (7)	C15—N2—S2—O3	171.8 (2)
C17—C16—C21—C20	0.4 (6)	C22—N2—S2—O4	−175.7 (3)
S2—C16—C21—C20	177.0 (3)	C15—N2—S2—O4	42.8 (3)
C27—C22—C23—C24	1.3 (6)	C22—N2—S2—C16	69.2 (3)
N2—C22—C23—C24	−179.0 (3)	C15—N2—S2—C16	−72.3 (3)
C22—C23—C24—C25	−2.4 (6)	C17—C16—S2—O3	−153.9 (3)
C23—C24—C25—C26	1.5 (6)	C21—C16—S2—O3	29.4 (4)
C23—C24—C25—Br1	178.5 (3)	C17—C16—S2—O4	−22.0 (4)
C24—C25—C26—O5	179.8 (4)	C21—C16—S2—O4	161.3 (3)
Br1—C25—C26—O5	2.8 (5)	C17—C16—S2—N2	92.0 (3)
C24—C25—C26—C27	0.5 (6)	C21—C16—S2—N2	−84.6 (3)

### N-{[3-Bromo-1-(phenylsulfonyl)-1H-indol-2-yl]methyl}-N-(4-bromo-3-methoxyphenyl)benzenesulfonamide (I) Hydrogen-bond geometry (Å, º)

**Table:**

*D*—H···*A*	*D*—H	H···*A*	*D*···*A*	*D*—H···*A*
C2—H2···O1	0.93	2.39	2.908 (5)	115
C11—H11···O4^i^	0.93	2.76	3.503 (6)	137
C15—H15*B*···O2	0.97	2.31	2.886 (4)	117
C18—H18···*Cg*(N1/C1/C6–C8)^ii^	0.93	2.99	3.861 (8)	156
C18—H18···*Cg*(C1–C6)^ii^	0.93	2.81	3.579 (1)	141

Symmetry codes: (i) *x*+1, *y*, *z*; (ii) *x*−1, *y*, *z*.

### N,N-Bis{[3-bromo-1-(phenylsulfonyl)-1H-indol-2-yl]methyl}benzenesulfonamide (II) Crystal data

**Table:**

C_36_H_27_Br_2_N_3_O_6_S_3_	*F*(000) = 1720
*M**_r_* = 853.60	*D*_x_ = 1.617 Mg m^−^^3^
Monoclinic, *P*2_1_/*n*	Mo *K*α radiation, λ = 0.71073 Å
*a* = 8.2664 (4) Å	Cell parameters from 73921 reflections
*b* = 34.7886 (18) Å	θ = 1.4–25.0°
*c* = 12.5972 (6) Å	µ = 2.54 mm^−^^1^
β = 104.550 (2)°	*T* = 303 K
*V* = 3506.5 (3) Å^3^	Block, colorless
*Z* = 4	0.29 × 0.19 × 0.04 mm

### N,N-Bis{[3-bromo-1-(phenylsulfonyl)-1H-indol-2-yl]methyl}benzenesulfonamide (II) Data collection

**Table:**

Bruker D8 Venture Diffractometer	5196 reflections with *I* > 2σ(*I*)
Radiation source: micro focus sealed tube	*R*_int_ = 0.071
ω and φ scans	θ_max_ = 25.5°, θ_min_ = 2.7°
Absorption correction: multi-scan (*SADABS*; Krause *et al.*, 2015)	*h* = −9→9
*T*_min_ = 0.491, *T*_max_ = 0.745	*k* = −41→41
73921 measured reflections	*l* = −15→15
6434 independent reflections	

### N,N-Bis{[3-bromo-1-(phenylsulfonyl)-1H-indol-2-yl]methyl}benzenesulfonamide (II) Refinement

**Table:**

Refinement on *F*^2^	0 restraints
Least-squares matrix: full	Hydrogen site location: inferred from neighbouring sites
*R*[*F*^2^ > 2σ(*F*^2^)] = 0.048	H-atom parameters constrained
*wR*(*F*^2^) = 0.120	*w* = 1/[σ^2^(*F*_o_^2^) + (0.0397*P*)^2^ + 5.0983*P*] where *P* = (*F*_o_^2^ + 2*F*_c_^2^)/3
*S* = 1.12	(Δ/σ)_max_ = 0.001
6434 reflections	Δρ_max_ = 0.46 e Å^−^^3^
451 parameters	Δρ_min_ = −0.47 e Å^−^^3^

### N,N-Bis{[3-bromo-1-(phenylsulfonyl)-1H-indol-2-yl]methyl}benzenesulfonamide (II) Special details

**Table:**

Geometry. All esds (except the esd in the dihedral angle between two l.s. planes) are estimated using the full covariance matrix. The cell esds are taken into account individually in the estimation of esds in distances, angles and torsion angles; correlations between esds in cell parameters are only used when they are defined by crystal symmetry. An approximate (isotropic) treatment of cell esds is used for estimating esds involving l.s. planes.

### N,N-Bis{[3-bromo-1-(phenylsulfonyl)-1H-indol-2-yl]methyl}benzenesulfonamide (II) Fractional atomic coordinates and isotropic or equivalent isotropic displacement parameters (Å^2^)

**Table:**

	*x*	*y*	*z*	*U*_iso_*/*U*_eq_	
C1	0.2266 (5)	0.42112 (11)	0.6112 (3)	0.0474 (10)	
C2	0.2075 (6)	0.45836 (12)	0.5688 (4)	0.0691 (14)	
H2	0.242319	0.465117	0.506636	0.083*	
C3	0.1341 (8)	0.48456 (15)	0.6241 (6)	0.090 (2)	
H3	0.119704	0.509708	0.598397	0.108*	
C4	0.0814 (7)	0.47499 (17)	0.7155 (6)	0.091 (2)	
H4	0.032049	0.493682	0.749854	0.109*	
C5	0.1002 (5)	0.43816 (16)	0.7576 (4)	0.0700 (14)	
H5	0.065741	0.431822	0.820239	0.084*	
C6	0.1726 (5)	0.41075 (12)	0.7029 (3)	0.0482 (10)	
C7	0.2180 (5)	0.37117 (11)	0.7239 (3)	0.0432 (9)	
C8	0.2986 (4)	0.35808 (10)	0.6506 (3)	0.0361 (8)	
C9	0.0542 (5)	0.37495 (11)	0.3929 (3)	0.0431 (9)	
C10	−0.0441 (6)	0.40600 (13)	0.3488 (3)	0.0568 (11)	
H10	0.004062	0.429860	0.343575	0.068*	
C11	−0.2147 (7)	0.40109 (18)	0.3127 (4)	0.0713 (14)	
H11	−0.282247	0.421807	0.283294	0.086*	
C12	−0.2844 (6)	0.36617 (19)	0.3199 (4)	0.0747 (15)	
H12	−0.399556	0.363131	0.295081	0.090*	
C13	−0.1865 (6)	0.33531 (16)	0.3634 (4)	0.0711 (14)	
H13	−0.235668	0.311493	0.367608	0.085*	
C14	−0.0158 (6)	0.33944 (12)	0.4008 (4)	0.0564 (11)	
H14	0.050914	0.318655	0.430778	0.068*	
C15	0.3938 (5)	0.32126 (10)	0.6489 (3)	0.0416 (9)	
H15A	0.315178	0.300654	0.621888	0.050*	
H15B	0.464582	0.324063	0.598503	0.050*	
C16	0.6415 (5)	0.24696 (10)	0.6959 (3)	0.0429 (9)	
C17	0.8099 (6)	0.24045 (14)	0.7415 (4)	0.0619 (12)	
H17	0.853833	0.242415	0.816857	0.074*	
C18	0.9117 (7)	0.23100 (17)	0.6738 (6)	0.0870 (18)	
H18	1.024933	0.226412	0.703439	0.104*	
C19	0.8454 (10)	0.22842 (16)	0.5629 (6)	0.091 (2)	
H19	0.914182	0.222430	0.517049	0.109*	
C20	0.6786 (10)	0.23460 (17)	0.5192 (4)	0.094 (2)	
H20	0.634731	0.232593	0.443842	0.113*	
C21	0.5756 (7)	0.24368 (13)	0.5848 (4)	0.0671 (14)	
H21	0.462055	0.247598	0.554608	0.081*	
C22	0.6628 (5)	0.33072 (11)	0.7938 (3)	0.0455 (9)	
H22A	0.717555	0.322827	0.868007	0.055*	
H22B	0.732885	0.322749	0.746573	0.055*	
C23	0.6489 (4)	0.37330 (11)	0.7907 (3)	0.0390 (8)	
C24	0.6816 (5)	0.39717 (12)	0.7143 (3)	0.0454 (9)	
C25	0.6421 (5)	0.43593 (12)	0.7368 (3)	0.0462 (9)	
C26	0.6451 (6)	0.47079 (14)	0.6812 (4)	0.0646 (13)	
H26	0.682286	0.471908	0.617395	0.078*	
C27	0.5912 (7)	0.50320 (14)	0.7244 (5)	0.0746 (15)	
H27	0.592151	0.526653	0.689090	0.090*	
C28	0.5358 (7)	0.50180 (13)	0.8183 (5)	0.0706 (14)	
H28	0.501929	0.524470	0.845424	0.085*	
C29	0.5286 (5)	0.46829 (12)	0.8734 (4)	0.0541 (11)	
H29	0.487987	0.467571	0.935870	0.065*	
C30	0.5854 (5)	0.43506 (10)	0.8315 (3)	0.0415 (9)	
C31	0.8482 (5)	0.39173 (12)	1.0480 (3)	0.0443 (9)	
C32	0.9122 (6)	0.42849 (16)	1.0514 (4)	0.0699 (14)	
H32	0.841943	0.449515	1.030325	0.084*	
C33	1.0838 (8)	0.4333 (2)	1.0869 (5)	0.094 (2)	
H33	1.130676	0.457639	1.088526	0.113*	
C34	1.1843 (7)	0.4018 (3)	1.1197 (5)	0.098 (2)	
H34	1.299428	0.405079	1.142745	0.117*	
C35	1.1190 (7)	0.3657 (2)	1.1194 (5)	0.0891 (19)	
H35	1.188587	0.344814	1.144262	0.107*	
C36	0.9479 (6)	0.36050 (15)	1.0816 (4)	0.0664 (13)	
H36	0.901555	0.336052	1.079059	0.080*	
N1	0.3054 (4)	0.38863 (8)	0.5760 (2)	0.0400 (7)	
N2	0.4990 (4)	0.31064 (8)	0.7588 (2)	0.0389 (7)	
N3	0.5879 (4)	0.39605 (8)	0.8669 (2)	0.0375 (7)	
O1	0.3214 (4)	0.41488 (10)	0.3958 (3)	0.0727 (9)	
O2	0.3521 (4)	0.34514 (9)	0.4273 (2)	0.0566 (7)	
O3	0.3523 (4)	0.24801 (9)	0.7430 (3)	0.0728 (10)	
O4	0.6071 (5)	0.25830 (9)	0.8922 (2)	0.0659 (9)	
O5	0.5475 (4)	0.41314 (9)	1.0495 (2)	0.0549 (7)	
O6	0.5948 (4)	0.34548 (8)	1.0092 (2)	0.0606 (8)	
S1	0.27243 (13)	0.38060 (3)	0.43993 (8)	0.0467 (2)	
S2	0.51553 (13)	0.26365 (3)	0.78055 (9)	0.0470 (2)	
S3	0.63083 (12)	0.38513 (3)	1.00122 (8)	0.0416 (2)	
Br1	0.74777 (6)	0.38250 (2)	0.58953 (4)	0.07276 (18)	
Br2	0.17344 (7)	0.34427 (2)	0.84137 (4)	0.07746 (19)	

### N,N-Bis{[3-bromo-1-(phenylsulfonyl)-1H-indol-2-yl]methyl}benzenesulfonamide (II) Atomic displacement parameters (Å^2^)

**Table:**

	*U*^11^	*U*^22^	*U*^33^	*U*^12^	*U*^13^	*U*^23^
C1	0.042 (2)	0.038 (2)	0.052 (2)	0.0029 (17)	−0.0057 (18)	−0.0091 (18)
C2	0.078 (3)	0.040 (2)	0.071 (3)	0.008 (2)	−0.016 (3)	0.001 (2)
C3	0.091 (4)	0.044 (3)	0.110 (5)	0.023 (3)	−0.022 (4)	−0.021 (3)
C4	0.060 (3)	0.073 (4)	0.119 (5)	0.031 (3)	−0.019 (3)	−0.050 (4)
C5	0.045 (3)	0.083 (4)	0.076 (3)	0.017 (2)	0.004 (2)	−0.031 (3)
C6	0.0325 (19)	0.055 (2)	0.052 (2)	0.0094 (17)	0.0005 (17)	−0.0170 (19)
C7	0.036 (2)	0.054 (2)	0.038 (2)	0.0044 (17)	0.0058 (16)	−0.0004 (17)
C8	0.0357 (19)	0.0359 (19)	0.0353 (19)	0.0020 (15)	0.0061 (15)	0.0013 (15)
C9	0.051 (2)	0.046 (2)	0.0330 (19)	0.0019 (18)	0.0108 (17)	−0.0048 (16)
C10	0.067 (3)	0.058 (3)	0.040 (2)	0.007 (2)	0.004 (2)	0.0027 (19)
C11	0.071 (3)	0.091 (4)	0.044 (3)	0.025 (3)	0.000 (2)	−0.005 (2)
C12	0.052 (3)	0.111 (5)	0.058 (3)	0.001 (3)	0.007 (2)	−0.027 (3)
C13	0.065 (3)	0.080 (4)	0.068 (3)	−0.024 (3)	0.018 (3)	−0.024 (3)
C14	0.066 (3)	0.048 (2)	0.052 (3)	−0.004 (2)	0.008 (2)	−0.0083 (19)
C15	0.046 (2)	0.0311 (19)	0.046 (2)	0.0052 (16)	0.0086 (17)	−0.0013 (16)
C16	0.053 (2)	0.0288 (19)	0.045 (2)	0.0068 (16)	0.0103 (18)	0.0000 (16)
C17	0.051 (3)	0.071 (3)	0.061 (3)	0.007 (2)	0.009 (2)	−0.014 (2)
C18	0.057 (3)	0.092 (4)	0.116 (5)	0.008 (3)	0.030 (3)	−0.021 (4)
C19	0.134 (6)	0.070 (4)	0.093 (5)	0.018 (4)	0.075 (4)	0.003 (3)
C20	0.152 (6)	0.083 (4)	0.049 (3)	0.058 (4)	0.029 (3)	−0.002 (3)
C21	0.089 (4)	0.051 (3)	0.054 (3)	0.025 (2)	0.006 (3)	−0.010 (2)
C22	0.039 (2)	0.046 (2)	0.050 (2)	0.0004 (17)	0.0076 (17)	−0.0103 (18)
C23	0.0342 (19)	0.044 (2)	0.0376 (19)	−0.0023 (15)	0.0062 (15)	−0.0092 (16)
C24	0.038 (2)	0.059 (3)	0.041 (2)	−0.0138 (18)	0.0134 (17)	−0.0082 (18)
C25	0.042 (2)	0.049 (2)	0.045 (2)	−0.0151 (18)	0.0055 (18)	−0.0016 (18)
C26	0.061 (3)	0.068 (3)	0.063 (3)	−0.021 (2)	0.012 (2)	0.015 (2)
C27	0.077 (3)	0.047 (3)	0.087 (4)	−0.014 (2)	−0.003 (3)	0.019 (3)
C28	0.077 (3)	0.039 (3)	0.089 (4)	−0.001 (2)	0.009 (3)	0.000 (2)
C29	0.055 (3)	0.045 (2)	0.057 (3)	0.0029 (19)	0.004 (2)	−0.007 (2)
C30	0.042 (2)	0.035 (2)	0.043 (2)	−0.0041 (16)	0.0021 (17)	−0.0016 (16)
C31	0.043 (2)	0.059 (3)	0.0315 (19)	−0.0041 (18)	0.0097 (16)	−0.0073 (17)
C32	0.058 (3)	0.083 (4)	0.060 (3)	−0.018 (3)	0.000 (2)	0.007 (3)
C33	0.072 (4)	0.132 (6)	0.070 (4)	−0.053 (4)	0.004 (3)	0.007 (4)
C34	0.042 (3)	0.196 (8)	0.058 (3)	−0.008 (4)	0.019 (3)	−0.018 (4)
C35	0.053 (3)	0.134 (6)	0.073 (4)	0.028 (4)	0.002 (3)	−0.033 (4)
C36	0.060 (3)	0.074 (3)	0.061 (3)	0.013 (2)	0.007 (2)	−0.020 (2)
N1	0.0451 (17)	0.0349 (16)	0.0385 (17)	0.0024 (13)	0.0079 (14)	−0.0004 (13)
N2	0.0416 (17)	0.0309 (16)	0.0441 (17)	0.0046 (13)	0.0106 (14)	0.0029 (13)
N3	0.0416 (17)	0.0360 (16)	0.0348 (16)	−0.0013 (13)	0.0095 (13)	−0.0033 (12)
O1	0.083 (2)	0.072 (2)	0.063 (2)	−0.0196 (18)	0.0183 (18)	0.0201 (17)
O2	0.0584 (18)	0.068 (2)	0.0465 (16)	0.0097 (15)	0.0196 (14)	−0.0085 (14)
O3	0.0582 (19)	0.0482 (18)	0.118 (3)	−0.0067 (15)	0.034 (2)	0.0129 (18)
O4	0.099 (3)	0.0575 (19)	0.0452 (17)	0.0221 (17)	0.0260 (17)	0.0143 (14)
O5	0.0543 (17)	0.0699 (19)	0.0461 (16)	0.0056 (14)	0.0228 (14)	−0.0075 (14)
O6	0.074 (2)	0.0516 (18)	0.0562 (18)	−0.0182 (15)	0.0162 (16)	0.0063 (14)
S1	0.0528 (6)	0.0515 (6)	0.0365 (5)	−0.0042 (5)	0.0126 (4)	0.0050 (4)
S2	0.0541 (6)	0.0357 (5)	0.0551 (6)	0.0062 (4)	0.0211 (5)	0.0076 (4)
S3	0.0416 (5)	0.0473 (5)	0.0378 (5)	−0.0050 (4)	0.0136 (4)	−0.0027 (4)
Br1	0.0661 (3)	0.1085 (4)	0.0535 (3)	−0.0273 (3)	0.0333 (2)	−0.0247 (3)
Br2	0.0688 (3)	0.1127 (5)	0.0610 (3)	0.0127 (3)	0.0352 (3)	0.0225 (3)

### N,N-Bis{[3-bromo-1-(phenylsulfonyl)-1H-indol-2-yl]methyl}benzenesulfonamide (II) Geometric parameters (Å, º)

**Table:**

C1—C6	1.387 (6)	C21—H21	0.9300
C1—C2	1.395 (6)	C22—C23	1.485 (5)
C1—N1	1.429 (5)	C22—N2	1.489 (5)
C2—C3	1.377 (8)	C22—H22A	0.9700
C2—H2	0.9300	C22—H22B	0.9700
C3—C4	1.371 (9)	C23—C24	1.348 (5)
C3—H3	0.9300	C23—N3	1.430 (4)
C4—C5	1.380 (8)	C24—C25	1.432 (6)
C4—H4	0.9300	C24—Br1	1.861 (4)
C5—C6	1.397 (6)	C25—C30	1.387 (6)
C5—H5	0.9300	C25—C26	1.405 (6)
C6—C7	1.434 (6)	C26—C27	1.374 (7)
C7—C8	1.347 (5)	C26—H26	0.9300
C7—Br2	1.864 (4)	C27—C28	1.373 (8)
C8—N1	1.429 (5)	C27—H27	0.9300
C8—C15	1.507 (5)	C28—C29	1.366 (6)
C9—C14	1.378 (6)	C28—H28	0.9300
C9—C10	1.381 (6)	C29—C30	1.400 (6)
C9—S1	1.763 (4)	C29—H29	0.9300
C10—C11	1.379 (7)	C30—N3	1.427 (5)
C10—H10	0.9300	C31—C36	1.365 (6)
C11—C12	1.357 (8)	C31—C32	1.381 (6)
C11—H11	0.9300	C31—S3	1.760 (4)
C12—C13	1.373 (8)	C32—C33	1.386 (7)
C12—H12	0.9300	C32—H32	0.9300
C13—C14	1.379 (7)	C33—C34	1.375 (10)
C13—H13	0.9300	C33—H33	0.9300
C14—H14	0.9300	C34—C35	1.366 (10)
C15—N2	1.485 (5)	C34—H34	0.9300
C15—H15A	0.9700	C35—C36	1.387 (7)
C15—H15B	0.9700	C35—H35	0.9300
C16—C21	1.373 (6)	C36—H36	0.9300
C16—C17	1.385 (6)	N1—S1	1.690 (3)
C16—S2	1.765 (4)	N2—S2	1.658 (3)
C17—C18	1.380 (7)	N3—S3	1.683 (3)
C17—H17	0.9300	O1—S1	1.417 (3)
C18—C19	1.370 (9)	O2—S1	1.426 (3)
C18—H18	0.9300	O3—S2	1.421 (3)
C19—C20	1.367 (9)	O4—S2	1.432 (3)
C19—H19	0.9300	O5—S3	1.416 (3)
C20—C21	1.364 (8)	O6—S3	1.420 (3)
C20—H20	0.9300		
			
C6—C1—C2	122.1 (4)	H22A—C22—H22B	107.7
C6—C1—N1	108.6 (3)	C24—C23—N3	107.8 (3)
C2—C1—N1	129.2 (4)	C24—C23—C22	127.3 (3)
C3—C2—C1	116.3 (6)	N3—C23—C22	124.9 (3)
C3—C2—H2	121.8	C23—C24—C25	110.0 (3)
C1—C2—H2	121.8	C23—C24—Br1	126.0 (3)
C4—C3—C2	122.5 (5)	C25—C24—Br1	123.7 (3)
C4—C3—H3	118.7	C30—C25—C26	120.0 (4)
C2—C3—H3	118.7	C30—C25—C24	107.0 (3)
C3—C4—C5	121.3 (5)	C26—C25—C24	132.9 (4)
C3—C4—H4	119.4	C27—C26—C25	117.5 (5)
C5—C4—H4	119.4	C27—C26—H26	121.2
C4—C5—C6	117.7 (6)	C25—C26—H26	121.2
C4—C5—H5	121.1	C28—C27—C26	121.6 (5)
C6—C5—H5	121.1	C28—C27—H27	119.2
C1—C6—C5	120.1 (4)	C26—C27—H27	119.2
C1—C6—C7	106.2 (3)	C29—C28—C27	122.3 (5)
C5—C6—C7	133.6 (5)	C29—C28—H28	118.8
C8—C7—C6	110.4 (4)	C27—C28—H28	118.8
C8—C7—Br2	127.2 (3)	C28—C29—C30	116.9 (5)
C6—C7—Br2	122.3 (3)	C28—C29—H29	121.6
C7—C8—N1	107.9 (3)	C30—C29—H29	121.6
C7—C8—C15	130.4 (3)	C25—C30—C29	121.6 (4)
N1—C8—C15	121.1 (3)	C25—C30—N3	107.9 (3)
C14—C9—C10	121.0 (4)	C29—C30—N3	130.3 (4)
C14—C9—S1	119.2 (3)	C36—C31—C32	122.1 (4)
C10—C9—S1	119.8 (3)	C36—C31—S3	119.1 (3)
C11—C10—C9	119.0 (5)	C32—C31—S3	118.8 (3)
C11—C10—H10	120.5	C31—C32—C33	118.3 (5)
C9—C10—H10	120.5	C31—C32—H32	120.9
C12—C11—C10	120.4 (5)	C33—C32—H32	120.9
C12—C11—H11	119.8	C34—C33—C32	119.6 (6)
C10—C11—H11	119.8	C34—C33—H33	120.2
C11—C12—C13	120.6 (5)	C32—C33—H33	120.2
C11—C12—H12	119.7	C35—C34—C33	121.6 (5)
C13—C12—H12	119.7	C35—C34—H34	119.2
C12—C13—C14	120.3 (5)	C33—C34—H34	119.2
C12—C13—H13	119.8	C34—C35—C36	119.2 (6)
C14—C13—H13	119.8	C34—C35—H35	120.4
C9—C14—C13	118.8 (4)	C36—C35—H35	120.4
C9—C14—H14	120.6	C31—C36—C35	119.2 (5)
C13—C14—H14	120.6	C31—C36—H36	120.4
N2—C15—C8	112.6 (3)	C35—C36—H36	120.4
N2—C15—H15A	109.1	C8—N1—C1	106.8 (3)
C8—C15—H15A	109.1	C8—N1—S1	121.4 (2)
N2—C15—H15B	109.1	C1—N1—S1	118.4 (3)
C8—C15—H15B	109.1	C15—N2—C22	115.8 (3)
H15A—C15—H15B	107.8	C15—N2—S2	113.9 (2)
C21—C16—C17	120.6 (4)	C22—N2—S2	112.5 (2)
C21—C16—S2	120.0 (3)	C30—N3—C23	107.2 (3)
C17—C16—S2	119.2 (3)	C30—N3—S3	120.8 (2)
C18—C17—C16	119.3 (5)	C23—N3—S3	121.9 (2)
C18—C17—H17	120.4	O1—S1—O2	119.9 (2)
C16—C17—H17	120.4	O1—S1—N1	105.65 (19)
C19—C18—C17	119.7 (5)	O2—S1—N1	107.17 (16)
C19—C18—H18	120.1	O1—S1—C9	109.3 (2)
C17—C18—H18	120.1	O2—S1—C9	109.17 (18)
C20—C19—C18	120.3 (5)	N1—S1—C9	104.47 (17)
C20—C19—H19	119.8	O3—S2—O4	120.0 (2)
C18—C19—H19	119.8	O3—S2—N2	106.90 (17)
C21—C20—C19	120.8 (5)	O4—S2—N2	106.97 (18)
C21—C20—H20	119.6	O3—S2—C16	109.1 (2)
C19—C20—H20	119.6	O4—S2—C16	107.72 (19)
C20—C21—C16	119.3 (5)	N2—S2—C16	105.13 (17)
C20—C21—H21	120.4	O5—S3—O6	120.34 (19)
C16—C21—H21	120.4	O5—S3—N3	105.89 (16)
C23—C22—N2	113.7 (3)	O6—S3—N3	107.18 (17)
C23—C22—H22A	108.8	O5—S3—C31	109.42 (18)
N2—C22—H22A	108.8	O6—S3—C31	108.5 (2)
C23—C22—H22B	108.8	N3—S3—C31	104.31 (17)
N2—C22—H22B	108.8		
			
C6—C1—C2—C3	1.0 (6)	C32—C33—C34—C35	−0.7 (9)
N1—C1—C2—C3	−176.3 (4)	C33—C34—C35—C36	2.2 (9)
C1—C2—C3—C4	−0.3 (8)	C32—C31—C36—C35	−0.6 (7)
C2—C3—C4—C5	0.3 (9)	S3—C31—C36—C35	−179.4 (4)
C3—C4—C5—C6	−0.9 (8)	C34—C35—C36—C31	−1.6 (8)
C2—C1—C6—C5	−1.7 (6)	C7—C8—N1—C1	1.3 (4)
N1—C1—C6—C5	176.1 (3)	C15—C8—N1—C1	−170.3 (3)
C2—C1—C6—C7	−178.1 (4)	C7—C8—N1—S1	−138.7 (3)
N1—C1—C6—C7	−0.4 (4)	C15—C8—N1—S1	49.7 (4)
C4—C5—C6—C1	1.6 (6)	C6—C1—N1—C8	−0.5 (4)
C4—C5—C6—C7	176.9 (4)	C2—C1—N1—C8	177.0 (4)
C1—C6—C7—C8	1.2 (4)	C6—C1—N1—S1	140.9 (3)
C5—C6—C7—C8	−174.6 (4)	C2—C1—N1—S1	−41.6 (5)
C1—C6—C7—Br2	179.2 (3)	C8—C15—N2—C22	−80.9 (4)
C5—C6—C7—Br2	3.4 (6)	C8—C15—N2—S2	146.3 (3)
C6—C7—C8—N1	−1.6 (4)	C23—C22—N2—C15	55.1 (4)
Br2—C7—C8—N1	−179.4 (3)	C23—C22—N2—S2	−171.5 (3)
C6—C7—C8—C15	169.0 (4)	C25—C30—N3—C23	0.3 (4)
Br2—C7—C8—C15	−8.8 (6)	C29—C30—N3—C23	176.2 (4)
C14—C9—C10—C11	0.2 (6)	C25—C30—N3—S3	146.3 (3)
S1—C9—C10—C11	−179.8 (3)	C29—C30—N3—S3	−37.7 (5)
C9—C10—C11—C12	−0.4 (7)	C24—C23—N3—C30	0.2 (4)
C10—C11—C12—C13	0.2 (8)	C22—C23—N3—C30	−176.6 (3)
C11—C12—C13—C14	0.2 (8)	C24—C23—N3—S3	−145.4 (3)
C10—C9—C14—C13	0.2 (6)	C22—C23—N3—S3	37.8 (5)
S1—C9—C14—C13	−179.8 (3)	C8—N1—S1—O1	−168.6 (3)
C12—C13—C14—C9	−0.5 (7)	C1—N1—S1—O1	55.9 (3)
C7—C8—C15—N2	−41.2 (5)	C8—N1—S1—O2	−39.6 (3)
N1—C8—C15—N2	128.3 (3)	C1—N1—S1—O2	−175.2 (3)
C21—C16—C17—C18	0.7 (7)	C8—N1—S1—C9	76.1 (3)
S2—C16—C17—C18	−173.8 (4)	C1—N1—S1—C9	−59.4 (3)
C16—C17—C18—C19	0.4 (8)	C14—C9—S1—O1	163.0 (3)
C17—C18—C19—C20	−1.0 (9)	C10—C9—S1—O1	−17.0 (4)
C18—C19—C20—C21	0.5 (10)	C14—C9—S1—O2	30.0 (4)
C19—C20—C21—C16	0.6 (9)	C10—C9—S1—O2	−150.0 (3)
C17—C16—C21—C20	−1.2 (7)	C14—C9—S1—N1	−84.3 (3)
S2—C16—C21—C20	173.2 (4)	C10—C9—S1—N1	95.7 (3)
N2—C22—C23—C24	−103.1 (4)	C15—N2—S2—O3	−45.1 (3)
N2—C22—C23—N3	73.1 (5)	C22—N2—S2—O3	−179.5 (3)
N3—C23—C24—C25	−0.6 (4)	C15—N2—S2—O4	−174.9 (3)
C22—C23—C24—C25	176.1 (3)	C22—N2—S2—O4	50.7 (3)
N3—C23—C24—Br1	−174.8 (3)	C15—N2—S2—C16	70.8 (3)
C22—C23—C24—Br1	1.9 (6)	C22—N2—S2—C16	−63.6 (3)
C23—C24—C25—C30	0.7 (4)	C21—C16—S2—O3	39.1 (4)
Br1—C24—C25—C30	175.1 (3)	C17—C16—S2—O3	−146.4 (3)
C23—C24—C25—C26	−176.4 (4)	C21—C16—S2—O4	170.9 (4)
Br1—C24—C25—C26	−2.0 (6)	C17—C16—S2—O4	−14.6 (4)
C30—C25—C26—C27	0.3 (6)	C21—C16—S2—N2	−75.3 (4)
C24—C25—C26—C27	177.2 (4)	C17—C16—S2—N2	99.2 (4)
C25—C26—C27—C28	−0.1 (7)	C30—N3—S3—O5	40.2 (3)
C26—C27—C28—C29	−1.0 (8)	C23—N3—S3—O5	−178.7 (3)
C27—C28—C29—C30	1.8 (7)	C30—N3—S3—O6	169.8 (3)
C26—C25—C30—C29	0.6 (6)	C23—N3—S3—O6	−49.1 (3)
C24—C25—C30—C29	−177.0 (4)	C30—N3—S3—C31	−75.2 (3)
C26—C25—C30—N3	177.0 (3)	C23—N3—S3—C31	65.8 (3)
C24—C25—C30—N3	−0.6 (4)	C36—C31—S3—O5	134.2 (3)
C28—C29—C30—C25	−1.6 (6)	C32—C31—S3—O5	−44.7 (4)
C28—C29—C30—N3	−177.1 (4)	C36—C31—S3—O6	1.1 (4)
C36—C31—C32—C33	2.0 (7)	C32—C31—S3—O6	−177.8 (3)
S3—C31—C32—C33	−179.1 (4)	C36—C31—S3—N3	−112.9 (3)
C31—C32—C33—C34	−1.4 (8)	C32—C31—S3—N3	68.2 (4)

### N,N-Bis{[3-bromo-1-(phenylsulfonyl)-1H-indol-2-yl]methyl}benzenesulfonamide (II) Hydrogen-bond geometry (Å, º)

**Table:**

*D*—H···*A*	*D*—H	H···*A*	*D*···*A*	*D*—H···*A*
C2—H2···O1	0.93	2.43	2.993 (7)	119
C13—H13···O3^i^	0.93	2.80	3.324 (6)	117
C14—H14···O4^i^	0.93	2.78	3.558 (5)	142
C15—H15*B*···O2	0.97	2.25	2.850 (5)	119
C18—H18···O3^ii^	0.93	2.73	3.575 (6)	151
C19—H19···O4^iii^	0.93	2.59	3.443 (6)	152
C19—H19···O6^iii^	0.93	2.81	3.467 (6)	129
C20—H20···Br2^iii^	0.93	3.02	3.536 (5)	117
C22—H22*A*···O6	0.97	2.39	2.950 (5)	116
C27—H27···O1^iv^	0.93	2.48	3.389 (6)	164
C28—H28···O5^v^	0.93	2.63	3.547 (6)	170
C29—H29···O5	0.93	2.35	2.907 (5)	118
C34—H34···O5^ii^	0.93	2.62	3.360 (6)	137
C35—H35···*Cg*(C16–C21)^vi^	0.93	2.91	3.729 (7)	147

Symmetry codes: (i) *x*−1/2, −*y*+1/2, *z*−1/2; (ii) *x*+1, *y*, *z*; (iii) *x*+1/2, −*y*+1/2, *z*−1/2; (iv) −*x*+1, −*y*+1, −*z*+1; (v) −*x*+1, −*y*+1, −*z*+2; (vi) *x*−1/2, −*y*−1/2, *z*−1/2.
